# Fly casting with ligand sliding and orientational selection supporting complex formation of a GPCR and a middle sized flexible molecule

**DOI:** 10.1038/s41598-022-17920-7

**Published:** 2022-08-13

**Authors:** Junichi Higo, Kota Kasahara, Gert-Jan Bekker, Benson Ma, Shun Sakuraba, Shinji Iida, Narutoshi Kamiya, Ikuo Fukuda, Hidetoshi Kono, Yoshifumi Fukunishi, Haruki Nakamura

**Affiliations:** 1 Graduate School of Information Science, University of Hyogo, 7-1-28 Minatojima Minamimachi, Chuo-ku, Kobe, Hyogo 650-0047 Japan; 2 Research Organization of Science and Technology, Ritsumeikan University, 1-1-1 Noji-higashi, Kusatsu, Shiga 525-8577 Japan; 3 College of Life Sciences, Ritsumeikan University, 1-1-1 Noji-higashi, Kusatsu, Shiga 525-8577 Japan; 4 Institute for Protein Research, Osaka University, 3-2 Yamada-oka, Suita, Osaka 565-0871 Japan; 5 Institute for Quantum Life Science, National Institutes for Quantum Science and Technology, 8-1-7 Umemi-dai, Kizu, Kyoto 619-0215 Japan; 6 Technology Research Association for Next-Generation Natural Products Chemistry, 2-3-26, Aomi, Koto-ku, Tokyo 135-0064 Japan; 7 Cellular and Molecular Biotechnology Research Institute, National Institute of Advanced Industrial Science and Technology (AIST), 2-3-26, Aomi, Koto-ku, Tokyo 135-0064 Japan; 8 Present Address: Artificial Intelligence Research Center, National Institute of Advanced Industrial Science and Technology (AIST), 2-4-7, Aomi, Koto-ku, Tokyo 135-0064 Japan

**Keywords:** Biophysics, Computational biology and bioinformatics

## Abstract

A GA-guided multidimensional virtual-system coupled molecular dynamics (GA-mD-VcMD) simulation was conducted to elucidate binding mechanisms of a middle-sized flexible molecule, bosentan, to a GPCR protein, human endothelin receptor type B (hETB). GA-mD-VcMD is a generalized ensemble method that produces a free-energy landscape of the ligand-receptor binding by searching large-scale motions accompanied with stable maintenance of the fragile cell-membrane structure. All molecular components (bosentan, hETB, membrane, and solvent) were represented with an all-atom model. Then sampling was conducted from conformations where bosentan was distant from the binding site in the hETB binding pocket. The deepest basin in the resultant free-energy landscape was assigned to native-like complex conformation. The following binding mechanism was inferred. First, bosentan fluctuating randomly in solution is captured using a tip region of the flexible N-terminal tail of hETB via nonspecific attractive interactions (fly casting). Bosentan then slides occasionally from the tip to the root of the N-terminal tail (ligand–sliding). During this sliding, bosentan passes the gate of the binding pocket from outside to inside of the pocket with an accompanying rapid reduction of the molecular orientational variety of bosentan (orientational selection). Last, in the pocket, ligand–receptor attractive native contacts are formed. Eventually, the native-like complex is completed. The bosentan-captured conformations by the tip-region and root-region of the N-terminal tail correspond to two basins in the free-energy landscape. The ligand-sliding corresponds to overcoming of a free-energy barrier between the basins.

## Introduction

So-called G protein-coupled receptors (GPCRs) are membrane proteins constructing a large evolutionarily related protein family with various molecular functions^1^. Generally speaking, GPCRs have seven transmembrane helices (TM1–TM7) embedded in the membrane. GPCRs activate cellular responses via detection of molecules outside the cell. Because GPCRs are related to many diseases, they have been important targets for drugs^2^. Endothelin-1 (ET1) is a peptide (21 residues long) with a strong vasoconstrictor action discovered in humans^3^. To exert its activity, ET1 transmits signals by interacting with two GPCRs: endothelin type A (ETA)^4^ and endothelin type B (ETB)^5^ receptors. The complex structure of human endothelin type-B receptor (hETB) and ET1 was resolved using X-ray crystallography^6^. In this complex, ET1 is bound to a binding pocket of hETB on the cytoplasmic side.

Bosentan competes with ET1 when binding to human ETA (hETA) and human hETB (hETB). It inhibits ET1’s vasoconstriction effect as an antagonist^7–9^. The tertiary structure of the bosentan-hETB complex was solved using X-ray crystallography^10^. In this structure, bosentan binds to the binding pocket of hETB more deeply than ET1. However, interaction patterns between the C-terminal segment of ET1 and amino-acid residues in the binding pocket of hETB are similar to those in the bosentan–hETB complex.

**Figure 1:**
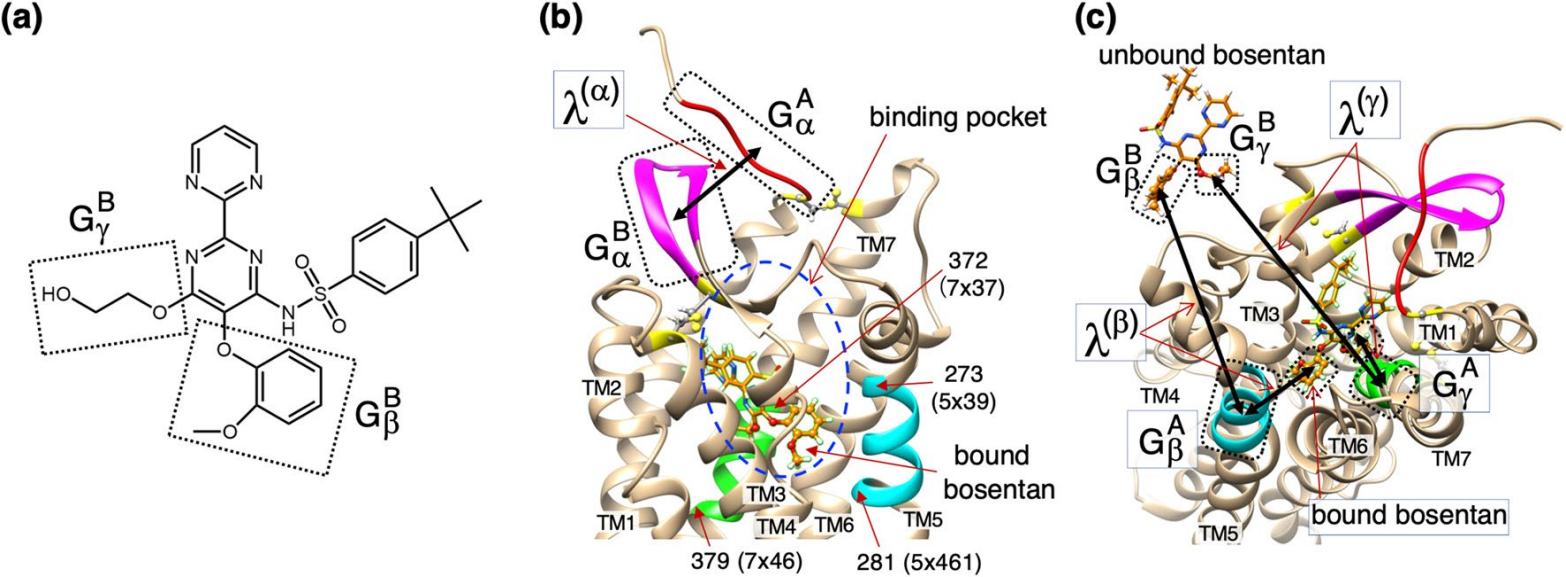
(**a**) Chemical structure of bosentan. Two atom groups $G_{\beta }^{B}$ and $G_{\gamma }^{B}$ are surrounded by dotted-line rectangles, which are used to set reaction coordinates (RCs) $\lambda^{\left( \beta \right)}$ and $\lambda^{\left( \gamma \right)}$. (**b**) The first RC, $\lambda^{\left( \alpha \right)}$, defined by distance between centroids of atom groups $G_{\alpha }^{A}$ (red segment in the N-terminal tail of hETB surrounded by dotted line; residues 85–89) and $G_{\alpha }^{B}$ (magenta-colored segment in $\beta$-hairpin of hETB surrounded by another dotted line; residues 243–254). Binding pocket of hETB is indicated by blue broken-line circle. Bound bosentan is shown at the bottom of the pocket. Green and cyan regions are defined in panel (**c**). (**c**) The second and third RCs are, respectively, $\lambda^{\left( \beta \right)}$ and $\lambda^{\left( \gamma \right)}$. Also, $\lambda^{\left( \beta \right)}$ is defined by the distance between centroids of $G_{\beta }^{A}$ (a cyan-colored part of the fifth transmembrane helix TM5; residues 273–281 (5 × 39–5 × 461), in which the first and last residues are indicated by “273(5 × 39)” and “281(5 × 461)”, respectively, in the panel) and $G_{\beta }^{B}$ (a part of bosentan surrounded by dotted line). $\lambda^{\left( \gamma \right)}$ is defined by $G_{\gamma }^{A}$ (a green part of the seventh transmembrane helix TM7; residues 372–379 (7 × 37–7 × 46), in which the first and last residues are indicated by “372(7 × 37)” and “379(7 × 46)”, respectively, in the panel) and $G_{\gamma }^{B}$ (other part of bosentan surrounded by the other dotted line). Bound and unbound bosentans are displayed. Yellow residues are cysteines that form disulfide bonds at the roots of the N-terminal tail, $G_{\alpha }^{A}$, and the $\beta$-hairpin, $G_{\alpha }^{B}$. Transmembrane helices (TM1–7) are labeled in panel **b**. Transmembrane helices (TM1–7) are labeled for panel (**b** and **c**).

Bosentan is a medium-sized (551.6 Da) drug molecule that is actually large compared to other commercial drug molecules. This molecule is flexible because it has long flexible sidechains (Fig. 1a), whereas the central ring, to which the sidechains are connected, is stiff. Consequently, bosentan will adopt temporal complex conformations (encounter complexes) before reaching the deep position of the hETB pocket (i.e., the native-complex position). A special molecular dynamics (MD) simulation that can sample various conformations in the complex-formation process is suitable for investigating those temporal conformations.

Recently, we introduced a generalized ensemble method, a multi-dimensional virtual-system coupled molecular dynamics (mD-VcMD) simulation^11^, which enhances conformational sampling with repetition of simulations (iteration) in a multi-dimensional (mD) reaction-coordinate (RC) space. By repeating the iterations, the sampled volume in the mD-RC space is expanded. The sampled conformations (snapshots) are then collected from the iterations. Importantly, mD-VcMD assigns a statistical weight (thermodynamic weight equilibrated at a simulation temperature) to each snapshot. Therefore, the ensemble of snapshots is equilibrated. Any equilibrated physicochemical quantity is computable from the ensemble if the quantity is expressed by the system’s coordinates. We designate this simulation method as the “original mD-VcMD method” or simply the “original method” in this paper. The original method was applied to a large and complicated system consisting of ET1 and hETB, where hETB was embedded in an explicit membrane and the membrane was immersed in an explicit solvent^12^. A benefit of mD-VcMD against other generalized ensemble methods is that RCs can be set arbitrarily to distances among atom groups. Consequently, this method can treat the molecular binding/unbinding process straightforwardly when RCs are set to distances between the two molecules (or between portions of the molecules). That is to say, enhancement of the motions of those RCs is related directly to molecular binding and unbinding.

Although the original mD-VcMD was applicable to the large and complicated system, we encountered an important difficulty: Once a poorly sampled region emerges in the mD-RC space in an iterative simulation, the conformation might be trapped in this region in subsequent iterations. Consequently, a more robust method is necessary to proceed with the sampling. To overcome this difficulty, we introduced a *genetic algorithm*-guided multi-dimensional virtual-system coupled molecular dynamics method (GA-mD-VcMD)^13^, which is an extension of the original mD-VcMD. We applied this method to some biological systems that consist of a protein and ligand in an explicit solvent^14–16^.

From the original mD-VcMD applied to binding of ET1 to hETB, two binding mechanisms were identified: Fly casting^17–19^ and conformational selection^20^. The receptor hETB has an intrinsically disordered N-terminal tail. The ET1 floating in solution was captured by the fluctuating N-terminal tail (fly casting mechanism) because the interactions between ET1 and the hETB’s N-terminal were attractive^12^. In fact, the root of the N-terminal tail is located at the gate of the binding pocket of hETB, which indicates that once ET1 is captured by the N-terminal tail, the ligand is restricted in a narrow volume around the gate of the binding pocket, resulting in an increased possibility that ET1 contacts the gate of the binding pocket. Next, only a fraction of various orientations of ET1 were allowed to enter the hETB pocket (conformational selection). This fractional entrance suggests that the pocket size is not sufficiently large for ET1 to rearrange the molecular orientation in the pocket, and that only ET1 with molecular orientations that are advantageous for the native complex formation is accepted.

For this study, we used GA-mD-VcMD simulation to sample interactions between the medium-sized molecule bosentan and hETB. Aside from the difference of ligand and simulation method, two differences exist between earlier studies and the present study. (1) We prolonged the intrinsically disordered N-terminal tail of hETB of the X-ray structure^6,10^, which were five and ten residues long for the earlier and the current studies, respectively^12^. This tail extension varies the features of the fly casting mechanism, as shown later. Because GPCRs have a long N-terminal tail in general^21^, this extension of the tail might support experimentation studies elucidating the role of the N-terminal tail of GPCRs^22,23^. Second, the initial simulation conformations for the present study were those in which bosentan was distant from hETB, whereas those for the previous study were the native-complex conformations of ET1 and hETB. In general, the difference of initial conformations influences the resultant conformational ensemble considerably. The reliability of findings obtained from the current study depends on the capacity of our method to sample native conformations. The native complex should be *discovered* as the lowest free-energy conformation out of many possible conformations. By contrast, in earlier studies, the native complex is always involved in the resultant ensemble, which means that the native complex might not necessarily correspond to the lowest free-energy conformation because a lower free-energy conformation might be overlooked in the sampling.

Our GA-mD-VcMD simulation produced a free-energy landscape of the bosentan–hETB system. Probability distribution functions of some quantities were calculated from the resultant conformational ensemble. Because the free-energy landscape covered both unbound conformations and the native-complex structures, and because the lowest free-energy conformation corresponded to the native-complex structure, we were able to analyze the complex formation comprehensively. We discuss the generality of the binding mechanism found in bosentan-hETB to ligand–GPCR binding. Because bosentan is a drug molecule, the present study helps drug development research not only by proposing the lowest free-energy conformations but also by investigating the binding process.

## Methods

The aim of this work is investigation of the complex-formation process of bosentan and hETB using GA-mD-VcMD method. First, we briefly explain this method, which provides a conformational ensemble of the system and a thermodynamic weight at the simulation temperature $T$ ($300 \,\mathrm{K}$ in this study), which is assigned to conformations stored in the ensemble. Details of GA-mD-VcMD have been explained elsewhere^13–16^. Next, we explain the molecular system which consists of hETB, bosentan, membrane (cholesterol and POPC lipid molecules), and solvent (water molecules and ions). Then, we describe multiple reaction coordinates that are introduced, along with the initial conformations used for simulation. Finally, we account for the method used to calculate the spatial density of bosentan around hETB.

We frequently use the term “native-like complex”. In general, an MD simulation is influenced by some force-field errors and sampling errors. Therefore, when a simulation is initiated from a completely unbound conformation, the simulation does not perfectly reproduce the experimentally observed conformation. For this reason, we designate the computed lowest free-energy conformation as “native-like complex conformation”, even when the conformation closely resembles the experimentally observed one.

However, we use the word “native complex conformation” for a computed conformation when a short simulation is done starting from the experimentally observed conformation because this simulation can be regarded as refinement of the experimentally observed conformation.

### GA-mD-VcMD and a conformational ensemble

To sample both the unbound and bound conformations of the system extensively, GA-mD-VcMD controls the system motions in a multi-dimensional (mD) space constructed by multiple reaction coordinates (RCs). The definition of an RC is presented in Section 1 of Supplementary Information (SI) and in Figure S1. We introduce three RCs denoted as ${\lambda }^{(h)}$ ($h=\alpha ,\beta,\,\mathrm{or} \,\gamma$), whereas the dimensionality of the RC space is arbitrary in theory^13^. Therefore, the word “multi-dimensional (mD)” designates three-dimensional (3D) for the present study. The actual definition for the three RCs is given later.

Conformational sampling of the system by GA-mD-VcMD at $300 \,\mathrm{K}$ produces a distribution (density) of the system in the mD-RC space (not in the real space). Because the conformational space of a biological molecular system is wide, the GA-mD-VcMD simulation is executed iteratively, by which the sampled volume of 3D-RC space is expanded. Here, the density at a 3D-RC position $[{\lambda }^{\left(\alpha \right)},{\lambda }^{\left(\beta \right)},{\lambda }^{\left(\gamma \right)}]$ is denoted as ${Q}_{cano}\left({\lambda }^{(\alpha )},{\lambda }^{(\beta )},{\lambda }^{(\gamma )}\right)$, with the density computed from the $M$-th iteration expressed as ${Q}_{cano}^{(M)}\left({\lambda }^{(\alpha )},{\lambda }^{(\beta )},{\lambda }^{(\gamma )}\right)$ when necessary.

The simulation is terminated when a convergence criterion is satisfied for ${Q}_{cano}\left({\lambda }^{(\alpha )},{\lambda }^{(\beta )},{\lambda }^{(\gamma )}\right)$. The convergence is discussed in the “Results and Discussion” section. Details of the convergence are discussed in Section 5 of SI. Snapshots of the system are saved from all iterations. After convergence, a thermodynamic weight at simulation temperature $T$ is assigned to each saved snapshot based on ${Q}_{cano}\left({\lambda }^{(\alpha )},{\lambda }^{(\beta )},{\lambda }^{(\gamma )}\right)$. Therefore, the snapshots construct a thermally equilibrated conformational ensemble (canonical ensemble) at $T$ ($300\, \mathrm{K})$.

### Molecular system studied

We generated the molecular system consisting of bosentan, hETB, cholesterols surrounding hETB, the POPC bilayer where hETB is embedded, and an explicit solvent (water molecules, ${\mathrm{Na}}^{+}$ and ${\mathrm{Cl}}^{-}$ ions). All of them were packed in a periodic boundary box. Details of system preparation are presented in Section 2 of SI. Figure S2 is a diagram outlining generation of the molecular system.

Two reference crystal structures are available for generating the molecular system for the simulation: The bosentan–hETB (PDB ID 5xpr; 3.6 Å resolution) and K8794–hETB (PDB ID 5x93; 2.2 Å) complex structures. Importantly, the two crystal structures are mutually similar to a considerable degree (Figure S3a) because K8794 is an analog of bosentan (Figure S3b). Because K8794–hETB complex has better resolution than bosentan–hETB complex, we used the K8794–hETB complex to prepare the molecular system.

First, we replaced K8794 with bosentan. This complex conformation is designated as “Complex structure of bosentan–hETB” in Figure S2. For crystallography, some mutations were applied to the amino-acid sequence of hETB to increase the diffraction data quality^10^. Then, we back-mutated these amino acids to those in the wild-type sequence of hETB. Finally, the computed hETB consists of residues 80–403. The N-terminal and C-termini of hETB were capped respectively by the acetyl and N-methyl groups denoted as Ace and Nme. Section 2 of SI presents details of system preparation.

Then the complex was embedded in the POPC bilayer. After four cholesterols were introduced into the hETB–membrane interface (Section 2 of SI), the system was immersed in a periodic boundary box filled with water molecules. The x-, y-, and z-coordinate axes for specifying the atomic positions were set parallel to the sides of the periodic box (the x–y plane was parallel to the membrane surface). The box dimensions were $71.33$ Å (x-axis), $71.33$ Å (y-axis), and $132.17$ Å (z-axis). Finally, sodium and chlorine ions were introduced by replacing water molecules randomly with ions to neutralize the system at a physiological ion concentration of 130 mM. This conformation is shown as the “Complex structure in periodic box” in Figure S2. The total number of atoms of the system was 69,062, with the numbers of protein and bosentan atoms as 5289 and 68, respectively, and with four cholesterols (296 atoms), 127 POPC molecules (17,018 atoms), 15,440 water molecules (46,320 atoms), and 28 sodium and 43 chlorine ions.

After energy minimization of the above system, with subsequent NVT and NPT simulations, we obtained the complex structure shown in Figure S4, for which the resultant periodic-box size was 69.37 Å × 69.37 Å × 140.29 Å. This structure is represented as “Complex structure in Figure S4 (native-complex structure)” in Figure S2. Because this complex structure is a relaxed conformation of the X-ray complex structure, bosentan was located at a position close to that in the X-ray structures (PDB IDs: 5xpr and 5x93). Therefore, we designate this conformation as the “native complex structure” in the present study. It is noteworthy that this native complex structure is *not* the initial conformation of the current GA-mD-VcMD simulations. As explained later, we generated conformations in which bosentan was separated completely from the binding pocket of hETB. They were the conformations that we used as the initial simulation conformations of GA-mD-VcMD.

The residue ordinal numbers used for this paper are based on two sources: the original PDB numbering (PDB ID 5xpr) and the GPCRdb generic numbering^24,25^. The GPCRdb generic residue numbers are presented in parentheses. When a residue is not numbered in the GPCRdb generic method, only the original PDB residue number is shown. For instance, residues of the N-terminal tail of hETB are not numbered in the GPCRdb generic method.

### Set of three RCs introduced to control system motions

Before describing generation of the initial conformations of GA-mD-VcMD, the set of three RCs (${\lambda }^{\left(h\right)}$; $h\in \{\alpha ,\beta ,\gamma \}$) introduced for the current study must be explained concretely. The setup of RCs is fundamentally important to increase the sampling efficiency, although RCs can be set arbitrarily in theory. We imposed three conditions on the RCs: the RCs can control (1) the gate opening and closing of the hETB binding pocket, (2) the ligand approaching to and departing from the receptor, and (3) the ligand orientation relative to the receptor.

Although various RCs can satisfy the three conditions presented above, we set them in a straightforward manner as shown in Fig. 1b,c. The exact specifications for the atom groups are given in the figure caption. We briefly explain the roles of the RCs. For condition (1), two atom groups ${G}_{\alpha }^{A}$ and ${G}_{\alpha }^{B}$ (Fig. 1b) are introduced to define the first RC ${\lambda }^{(\alpha )}$, where the increase and decrease of ${\lambda }^{(\alpha )}$ correspond to the opening and closing of the gate of the hETB’s binding pocket, respectively. We note that disulfide bonds are formed at the roots of the N-terminal tail, ${G}_{\alpha }^{A}$, and the $\beta$-hairpin, ${G}_{\alpha }^{B}$, as shown in Fig. 1b,c. This means that the roots of the N-terminal tail and $\beta$-hairpin are locked by the disulfide bonds and that other regions of hETB are only weakly influenced by the gate motions. Figure 1c shows atom groups ${G}_{\beta }^{A}$ and ${G}_{\beta }^{B}$ to define ${\lambda }^{(\beta )}$, as well as ${G}_{\gamma }^{A}$ and ${G}_{\gamma }^{B}$ to define ${\lambda }^{(\gamma )}$. Groups ${G}_{\beta }^{A}$ and ${G}_{\beta }^{B}$ are mutually close in the native complex; ${G}_{\gamma }^{A}$ and ${G}_{\gamma }^{B}$ are as well. Likewise, those atom groups are distant from each other in an unbound conformation. The increase and decrease of ${\lambda }^{(\beta )}$ and ${\lambda }^{(\gamma )}$ result in approaching and departing of bosentan to the hETB’s binding pocket: Condition (2) is established. Finally, we note that bosentan rotates with respect to hEETB when ${\lambda }^{(\beta )}$ decreases concomitantly with increasing ${\lambda }^{(\gamma )}$: Condition (3) is established. Figure 1a portrays a closeup image of bosentan to clarify ${G}_{\beta }^{B}$ and ${G}_{\gamma }^{B}$.

These atom groups are set as independent of the chemical properties of the ligand and receptor. The atom groups are set conveniently to establish the three conditions (1), (2), and (3) above. As described later, this set of RCs produced various gate–widths of the hETB’s binding pocket as well as various bosentan conformations relative to hETB.

One might infer that enhancement of the conformational motions in the 3D RC space affect the resultant conformational distribution and snapshots because a bias is introduced for the enhancement^13^. This possible effect of bias is true for conventional MD simulation. In GA-mD-VcMD, however, the bias effect is removed entirely from the resultant ensemble using a reweighting technique^13^.

### Initial conformations for GA-guided mD-VcMD

We explain generation of the initial conformations of GA-mD-VcMD. Our aim is to explore the conformational space from unbound to bound states. Consequently, the initial conformations should not be the native complex structure but should be a completely unbound conformation. Because we had only the native complex structure (Figure S4) at this stage, it was necessary to generate the unbound conformations from the complex structure. For this purpose, we performed ten simulations (called “separation simulations”) starting from the native complex structure with imposition of three requirements to RCs to the system’s conformation: 24.0 Å $\le {\lambda }^{\left(\alpha \right)}\le$ 25.0 Å, 60.0 Å $\le {\lambda }^{\left(\beta \right)}\le$ 65.0 Å, and 60.0 Å $\le {\lambda }^{\left(\gamma \right)}\le$ 65.0 Å. Ten separation simulations were run at 300 K for $5\times 1{0}^{6}$ steps, with a time step of 2 fs. The three RCs for the native complex structure are ${\lambda }^{(\alpha )}=8.22$ Å, ${\lambda }^{(\beta )}=8.35$ Å, and ${\lambda }^{(\gamma )}=6.21$ Å. During the separation simulations, bosentan moved from the binding pocket to unbound conformations. The resultant conformations are presented in Figure S5, where bosentans are separated completely from hETB, which are presented as “Unbound conformations in figure S5” in Figure S2.

Next, for additional relaxation of those unbound conformations, we performed 2000 runs: 200 ($1\times 1{0}^{6}$ steps with a time step of 2 fs at 300 K) runs starting from each of those ten unbound conformations with different random seeds to the initial atomic velocities. For the 2000 runs, we applied the following three restrictions to RCs: ${\lambda }^{\left(\alpha \right)}\le 25.0$ Å, ${\lambda }^{\left(\beta \right)}\le 65.0$ Å, and ${\lambda }^{\left(\gamma \right)}\le 65.0$ Å. The upper limits were introduced to prevent bosentan from flying further away from hETB in the periodic box. We refer to those 2000 runs as “diffusion simulations”. The last snapshots from the 2000 runs were then used as the initial conformations of the GA-mD-VcMD simulations. Figure S6 shows the positions of the last snapshots of the simulations in the 3D-RC space and the native complex structure in the 3D-RC space. Those conformations are represented as “Distributed conformations in figure S6” in figure S2.

It is noteworthy that Figure S6 demonstrates that the conformations sampled in the diffusion simulations did not reach the native complex structure. We emphasize that none of the current results was influenced by the structural features of the native complex because the 2000 initial conformations are those diffused freely from the ten completely unbound conformations (Figure S5).

### GA-mD-VcMD simulations

As described in the “Introduction” section, GA-mD-VcMD enhances conformational sampling in the 3D-RC space. The thermodynamic weights of sampled conformations at the simulation temperature (300 K) are computed using the converged distribution ${Q}_{cano}\left({\lambda }^{(\alpha )},{\lambda }^{(\beta )},{\lambda }^{(\gamma )}\right)$.

In the original mD-VcMD^12^ and GA-mD-VcMD^13^, each RC axis ${\lambda }^{\left(h\right)}$ ($h\in \{\alpha ,\beta ,\gamma \}$) is divided into small 1D zones (originally designated as “virtual zones”^13^). Accordingly, the 3D-RC space is divided into small 3D zones. Table S2 lists the actual division of each 1D axis into zones. During a short time-interval $\Delta {\tau }_{trns}$ of simulation, the system’s conformation freely moves only in a single 3D zone with a restoring force outside the zone. Then, an inter-zone transition from the zone to one of neighboring zones is attempted at every time interval. For the present study, we set $\Delta {\tau }_{trns}=20\,{\rm ps}$, which corresponds to $1\times {10}^{4}$ simulation steps, with a time step of $2\,{\rm fs}$. The inter-zone transition probabilities are determined using ${Q}_{cano}$^12,13^. In this way, the conformation moves in a wide 3D-RC space by inter-zone transitions.

We performed ${N}_{run}$ (= 2000) runs at $300\, \mathrm{K}$ in parallel starting from different conformations in each iteration to increase the sampling efficiency further. A simple integration of the ${N}_{run}$ trajectories can be regarded as a single, long trajectory by connecting the trajectories in arbitrary order^26,27^. The ${N}_{run}$ initial conformations for the first iteration of GA-mD-vcMD are the last conformations from the diffusion simulations (Figure S6). We obtained snapshots from the first to $M$-th iterations when the $M$-th iteration had finished. The ${N}_{run}$ initial conformations for the $(M+1)$-th iteration were selected from those snapshots so that they distribute as evenly as possible in the 3D-RC space. However, when a certain RC region was poorly sampled, we selected more snapshots from or near the poorly sampled RC region than from well-sampled RC regions^13^.

We set the simulation length of each run to $1\times {10}^{6}$ steps ($1\times {10}^{6}\times 2\,\mathrm{ fs}=2.0\,\,{ \mathrm{ns}}$). Consequently, the total length for an iteration was $4.0 {\upmu}s$ (= $2.0\,\mathrm{ ns}\times 2000$). We performed 55 iterations for the present study, which corresponds to $220.0\, \mathrm{\upmu s}$ ($4.0 \mathrm{\upmu s}\times 55$) in all. A snapshot was stored every $1\times {10}^{5}$ steps ($0.2 \,\mathrm{ns}$) in each run. Therefore, $1.1\times {10}^{6}$ ($220.0 \,\mathrm{\upmu}s/0.2 \,\mathrm{ns}$) snapshots were stored in all. We calculated the distribution functions of various quantities at the simulation temperature ($300\, \mathrm{K}$) from the resultant ensemble of snapshots.

The GA-mD-VcMD algorithm^13^, which was initially available in a MD simulation program omegagene/myPresto^28^, has been implemented for the current study in GROMACS^29^ using the PLUMED plug-in^30^. The simulation conditions were the following: LINCS^31^ to constrain the covalent-bond lengths related to hydrogen atoms, the Nosé–Hoover thermostat^32,33^ to control the simulation temperature, the zero-dipole summation method^34–36^ to compute the long-range electrostatic interactions accurately and quickly, a time-step of 2 fs ($\Delta t=2$ fs), and simulation temperature of $300\, {\mathrm{K}}$. All simulations were performed on the TSUBAME3.0 supercomputer at the Tokyo Institute of Technology using GP-GPU.

The force fields used are AMBER99SB-ILDN^37^ for hETB and AMBER LIPID14^38^ for the cholesterol and POPC lipid molecules, with TIP3P^39^ for water molecules and Joung–Cheatham model^40^ for ions (chloride and sodium), respectively. The bosentan force field was prepared as follows: First, the bosentan atomic partial charges were calculated quantum-chemically using Gaussian09^41^ at the HF/6-31G* level, followed by RESP fitting^42^. Then, the obtained partial charges were incorporated into a general amber force field (GAFF) force-field file^43^, which was designed to be compatible with conventional AMBER force-fields. The chemical structure of bosentan is shown in Fig. 1A.

### Spatial density of bosentan around hETB

GA-mD-VcMD simulation produces a canonical ensemble of conformations (snapshots) and assigns a thermodynamic weight ($300\, {\mathrm{K}}$) to each snapshot. Therefore, using the ensemble, one can calculate a spatial density of the bosentan centroid around hETB at 300 K using the ensemble. Details of methods used to calculate the spatial density are presented in Subsection 3.1 of SI.

We briefly explain the notation used to express the spatial density. The 3D real space (not the 3D-RC space) is divided into cubes (3 Å × 3 Å × 3 Å), with centers denoted as ${{\varvec{r}}}_{cube}$. The spatial density ${\rho }_{CMb}({{\varvec{r}}}_{cube})$ is the probability of detecting the bosentan centroid ${{\varvec{r}}}_{CMb}$ in the cube centered at ${{\varvec{r}}}_{cube}$.

## Results and discussion

We repeated 55 iterations of GA-mD-VcMD while saving $1.1\times {10}^{6}$ snapshots from the first to 55th iterations in all, as described in Methods. First, we show that the GA-mD-VcMD produced a distribution of the system that covered both the unbound and native-like complex conformations. We next show that the highest-density spot (the lowest free-energy spot) corresponds to the native-like complex structure. We then analyze the binding process from the unbound to intermediates, and from the intermediates to the native-like complex. For this purpose, the binding process is divided into three stages: the early stage of binding, where bosentan is outside the binding pocket; the second stage, where bosentan is passing the gate of the binding pocket; and the last stage, where bosentan is moving in the binding pocket.

### Conformational distribution in 3D-RC space

GA-mD-VcMD produced the distribution ${Q}_{cano}\left({\lambda }^{(\alpha )},{\lambda }^{(\beta )},{\lambda }^{(\gamma )}\right)$ equilibrated at $300\,{\rm K}$ in the 3D-RC space (Figure S8). Importantly, the distribution covered both the unbound and bound conformations. The native-complex structure was located at the periphery of the lowest free-energy basin. Section 4 of SI explains the features of this distribution.

The convergence check of the distribution is argued in Section 5 of SI with a function, ${E}_{local}({\lambda }^{\left(\alpha \right)},{\lambda }^{\left(\beta \right)},{\lambda }^{\left(\gamma \right)})$. This function was introduced originally in an earlier report of a study^13^. The value of this function is calculated at zones $({\lambda }^{\left(\alpha \right)},{\lambda }^{\left(\beta \right)},{\lambda }^{\left(\gamma \right)})$ in each iteration. Simply, when an inequality ${E}_{local}({\lambda }^{\left(\alpha \right)},{\lambda }^{\left(\beta \right)},{\lambda }^{\left(\gamma \right)})<0.25$ is satisfied at a zone, it is judged that ${Q}_{cano}$ is determined accurately at the zone. The validity of this threshold value has been checked repeatedly^14–16^.

Figure S9 shows that iterations 1–10 (Figures S9a–c) sampled a large volume of the 3D-RC space involving the unbound and bound conformations. Therefore, the simulation could be halted at the tenth iteration. However, to obtain snapshots sufficient for analysis, we continued the simulation up to the 55th iteration. A snapshot of every $0.2\,{ \mathrm{ns}}$ (100,000 steps) of the simulation was stored.

Next, we compared the distribution ${Q}_{cano}\left({\lambda }^{(\alpha )},{\lambda }^{(\beta )},{\lambda }^{(\gamma )}\right)$ with that obtained from an earlier study of the ET1–hETB system^12^. Section 6 of SI presents details of the comparative method. Figure S10 shows that the free-energy slope along the ${\lambda }^{(\alpha )}$ axis (the motion along the gate opening and closing of the hETB’s binding pocket) is gentler than those along the ${\lambda }^{(\beta )}$ or ${\lambda }^{(\gamma )}$ axis (bosentan’s approaching and departing motions to hETB). This result agrees with that obtained from the earlier study. However, the slope in the current bosentan–hETB landscape was about one-tenth of that in the previous ET1-hETB landscape.

From the earlier study^12^, we inferred that the steep slope is probably caused by a shortcoming of the update method of the probability distribution ${Q}_{cano}$ in the 3D RC space (i.e., ${Q}_{cano}^{(M)}\to {Q}_{cano}^{(M+1)}$) and that the steep slope would be weakened if two pieces of ${Q}_{cano}^{\left(M\right)}({\lambda }^{(\alpha )},{\lambda }^{(\beta )},{\lambda }^{(\gamma )})$ and ${Q}_{cano}^{\left(M\right)}({\lambda }^{\left(\alpha \right)}{^{\prime}},{\lambda }^{\left(\beta \right)}{^{\prime}},{\lambda }^{\left(\gamma \right)}{^{\prime}})$ were connected adequately, where $({\lambda }^{\left(\alpha \right)},{\lambda }^{\left(\beta \right)},{\lambda }^{\left(\gamma \right)})$ and $({{\lambda }^{\left(\alpha \right)}}^{^{\prime}},{{\lambda }^{\left(\beta \right)}}^{^{\prime}},{{\lambda }^{\left(\gamma \right)}}^{^{\prime}})$ are mutually neighboring zones in the RC space. GA-mD-VcMD provides such a method to connect ${Q}_{cano}^{(M)}$ smoothly^13^. In fact, the free-energy slope decreased in the present study significantly.

### Three-dimensional (3D) spatial distribution of bosentan around hETB

**Figure 2:**
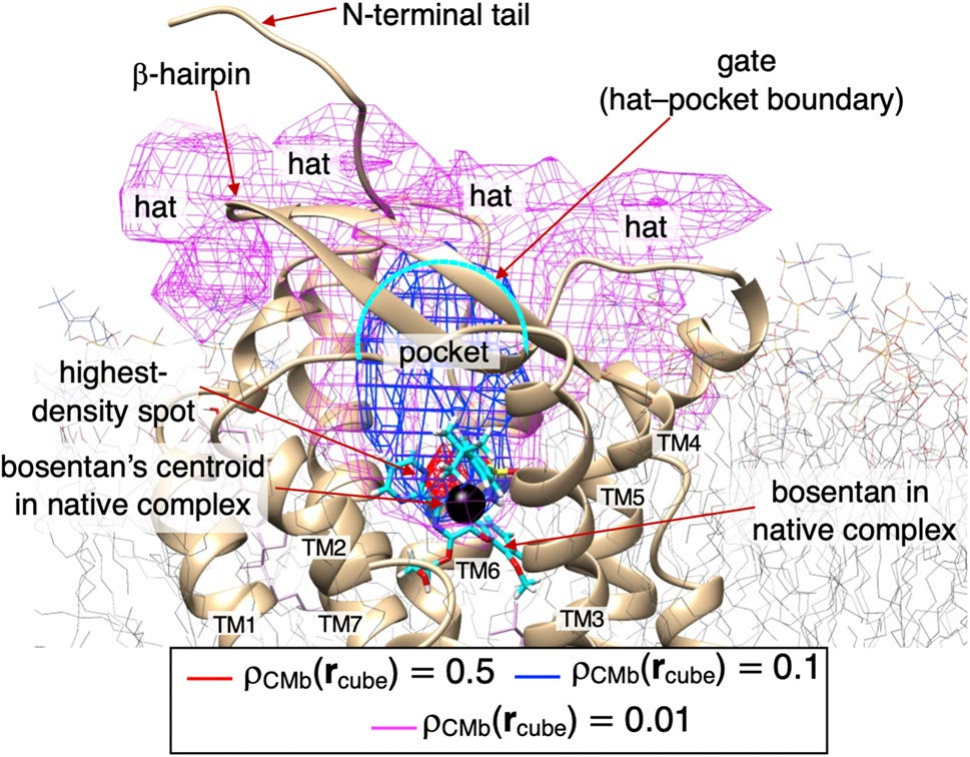
The spatial density $\rho_{CMb} \left( {{\varvec{r}}_{cube} } \right)$ of the bosentan centroid around hETB. The iso-density map is presented at three contour levels with different colors, as shown in the inset. The molecular structure is the native complex (Figure S4) omitting solvent. Bosentan is shown as a cyan-colored stick model, with its centroid shown as a small black sphere. N-terminal tail and $\beta$-hairpin of hETB are indicated with labels. Red-colored contours are named “highest-density spots”. Blue-colored ones ($\rho_{CMb} \left( {{\varvec{r}}_{cube} } \right) \ge 0.1$) well specify the binding pocket of hETB. Magenta-colored regions above the pocket are designated as “hat”. Cyan-colored broken-lines represent the boundary, the hat–pocket boundary, which is called “gate” of the binding pocket. Figure S11, presents $\rho_{CMb} \left( {{\varvec{r}}_{cube} } \right)$ with addition of two low-density contours of $\rho_{CMb} \left( {{\varvec{r}}_{cube} } \right) \ge 0.001$ and $0.0001$. Transmembrane helices (TM1–7) are labeled.

Whereas GA-mD-VcMD samples the mD-RC space, the conformational distribution in the mD-RC space does not always provide a concrete or intuitive image for the molecular conformation of the system. Therefore, we converted the distribution ${Q}_{cano}\left({\lambda }^{(\alpha )},{\lambda }^{(\beta )},{\lambda }^{(\gamma )}\right)$ (Figure S8) to the conformational distribution ${\rho }_{CMb}({{\varvec{r}}}_{cube})$ defined in the 3D real space using a thermodynamic weight assigned to all the sampled snapshots using Eq. 31 of that earlier study^13^. Subsection 3.1 of SI presents the method to calculate ${\rho }_{CMb}({{\varvec{r}}}_{cube})$. Figure 2 portrays the spatial patterns of ${\rho }_{CMb}({{\varvec{r}}}_{cube})$ of the bosentan centroid ${{\varvec{r}}}_{CMb}$ around hETB. Importantly, the highest-density spot (the red contours; ${\rho }_{CMb}({{\varvec{r}}}_{cube})\ge 0.5$) well agreed with the position of bosentan centroid in the native complex structure. This result is important because the reliability of the simulation data decreases unless the highest-density spot is assigned to the native bosentan position.

The binding pocket corresponds to the region of ${\rho }_{CMb}({{\varvec{r}}}_{cube})\ge 0.1$ (blue contours), which involved the highest-density spot. Outside of the binding pocket was characterized by a lower density of ${\rho }_{CMb}({{\varvec{r}}}_{cube})\ge 0.01$ (magenta-colored ones), which was named a “density hat” or simply “hat”. We denote the hat–pocket boundary (cyan-colored broken line in Fig. 2) as the “gate” of the binding pocket. We emphasize that the gate is not defined by residues of hETB but rather assigned to the boundary between the low- and high-density regions of ${\rho }_{CMb}({{\varvec{r}}}_{cube})$. In other words, the gate is a quantity that is dependent on the density field.

Figure S11 depicts the spatial density for the whole sampled region. Bosentan was distributed even in regions that are distant from hETB, as evidenced by the low density: ${\rho }_{CMb}({{\varvec{r}}}_{cube})\ge 0.0001$. The bosentan density increased rapidly with approach to the binding pocket from the far region. This figure demonstrates that GA-mD-VcMD is powerful for sampling the wide conformational space efficiently.

### RMSD of bosentan between the snapshot and the native complex

**Figure 3:**
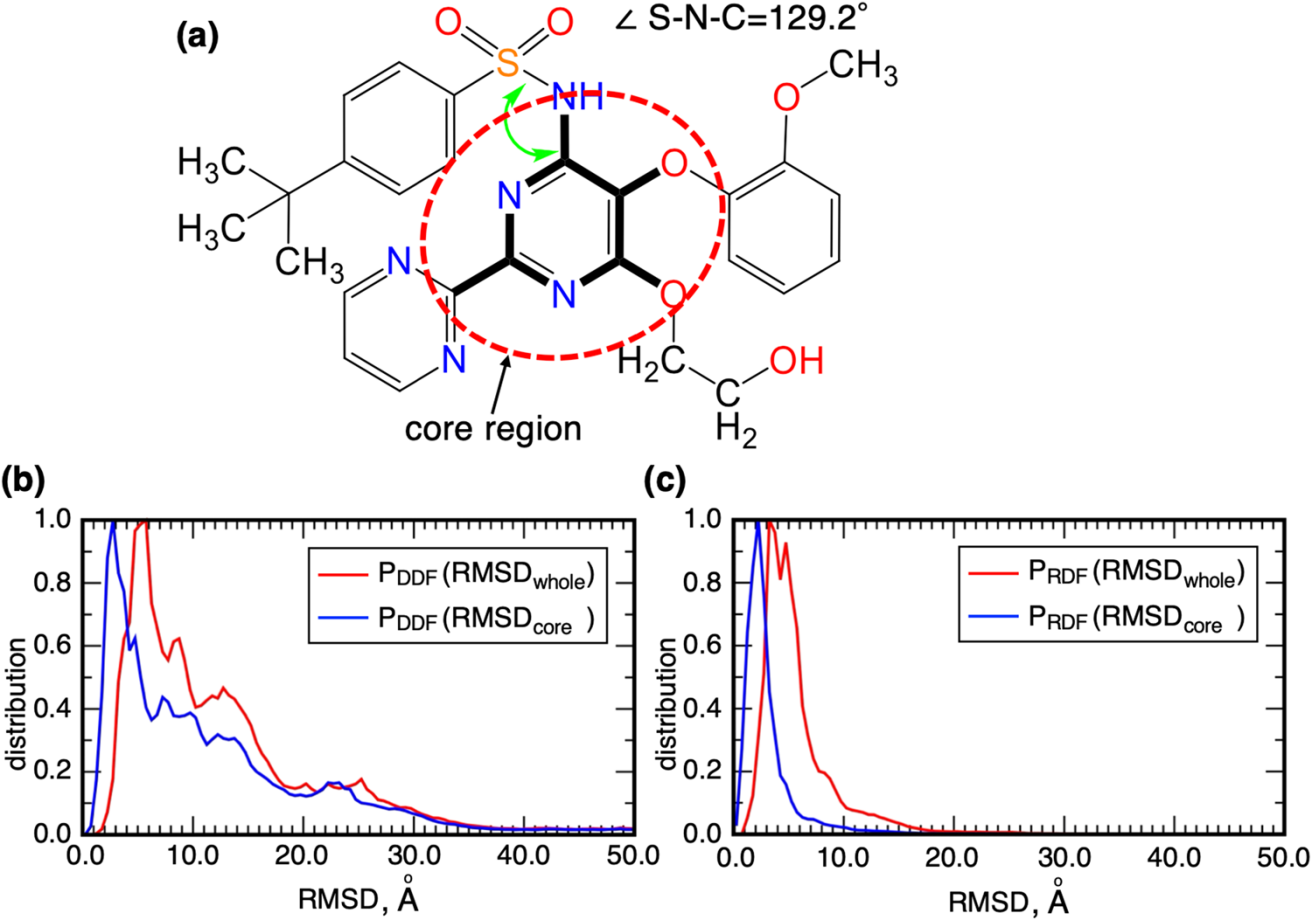
(**a**) Bosentan’s “core region”, which is surrounded by a red broken line. The core region located at the center of bosentan can be regarded approximately as a rigid body. Therefore, the core region is a representative part to express the bosentan position and orientation. (**b**) Distance distribution function $P_{DDF} \left( {RMSD} \right)$ and (**c**) radial distribution function $P_{RDF} \left( {RMSD} \right)$ are presented for both $RMSD_{whole}$ and $RMSD_{core}$. Definitions of $RMSD$, $P_{DDF}$, and $P_{RDF}$ are given in Subsection 3.2 of SI.

As presented above, we showed visual agreement of the computed highest-density spot and the bosentan native-complex position. Here, to analyze the agreement quantitatively, we calculated the root-mean-square-deviations $RMSD$ of bosentan of two types between a snapshot and the native complex: $RMSD={RMSD}_{whole}$ or $RMSD={RMSD}_{core}$. ${RMSD}_{whole}$ is calculated using all the heavy atoms in bosentan. Also, ${RMSD}_{core}$ is calculated using the heavy atoms in the bosentan core region. The definition of the core region is presented in Fig. 3a.

The ten heavy atoms comprising the core region are located on a plane and are connected firmly by covalent bonds. Consequently, this region can be regarded as a rigid body approximately. Furthermore, this region is located at the bosentan center surrounded by four sidechains. Therefore, the core region is a representative part for expressing the bosentan position and orientation.

We calculated two probability distribution functions to analyze $RMSD$: the distance distribution function ${P}_{DDF}(RMSD)$ and the radial distribution function ${P}_{RDF}(RMSD)$. Subsection 3.2 of SI presents details of ${P}_{DDF}$ and ${P}_{RDF}$. The resultant distributions are presented in Fig. 3. For the whole bosentan, the highest-peak positions of ${P}_{RDF}$ and ${P}_{DDF}$ are, respectively, at ${RMSD}_{whole}\approx 3.25$ Å and ${RMSD}_{whole}\approx 5.75$ Å. Similarly, for the core region, those are respectively at ${RMSD}_{core}\approx 2.25$ Å and ${RMSD}_{core}\approx 2.75$ Å. The peak positions for the core region were smaller than those for the whole bosentan because of the disorder of the longest sidechain of bosentan, as explained later.

### Bosentan–tail contact ratio

The structure of the N-terminal tail is not determined in the X-ray structures (PDB IDs: 5x93 and 5xpr) because the tail is intrinsically disordered. We presume that the tail might affect the binding process because the root of the tail is located at the entrance of the hETB binding pocket. Results of some experimentation studies have indicated that an extracellular loop of GPCRs plays a role as a lid to block the gate of the binding pocket^44,45^.

Figure S7b highlights the gate of the hETB binding pocket. As this figure shows, half of the gate, which is covered by the white solid-line rectangle, is closed by the contact of the N-terminal tail and the $\beta$-hairpin. The other half of the gate, which is covered by the white broken-line rectangle, is open. The definition of the N-terminal tail and the $\beta$-hairpin is given in the caption of Figure S7b. One can readily imagine from this figure that the gate situation can vary during tail fluctuations. Below, we investigate the tail fluctuations and the bosentan–tail contact.

First, to visualize fluctuations of the N-terminal tail, we calculated a spatial density function ${\rho }_{CMNt}({{\varvec{r}}}_{cube})$ for the N-terminal tail tip. We also calculated the spatial density function ${\rho }_{CM\beta h}({{\varvec{r}}}_{cube})$ for the tip of the $\beta$-hairpin because the $\beta$-hairpin is the contact partner of the N-terminal tail to open and close the gate of the binding pocket. Figure S12a exhibits that the N-terminal tail fluctuates widely with varying its orientation, which is a natural property of the intrinsically disordered segment. In contrast, the $\beta$-hairpin fluctuation is highly limited in a narrow volume, as shown in Figure S12b. Therefore, the gate opening and closing is induced by the large motions of the N-terminal tail (not by the fluctuation of $\beta$-hairpin).

Next, to investigate the bosentan–tail contact, we introduced a “cube-based bosentan–tail contact ratio” denoted as ${\overline{c} }_{b-N}({{\varvec{r}}}_{cube})$. It is noteworthy the bosentan centroid ${{\varvec{r}}}_{CMb}$ in a snapshot was assigned to a cube at ${{\varvec{r}}}_{cube}$, which involved ${{\varvec{r}}}_{CMb}$. Then ${\overline{c} }_{b-N}({{\varvec{r}}}_{cube})$ is the ratio by which bosentan in ${{\varvec{r}}}_{cube}$ contacts to the N-terminal tail. The maximum and minimum of ${\overline{c} }_{b-N}({{\varvec{r}}}_{cube})$ are, respectively, 1.0 and 0.0. Detailed procedures used to calculate ${\overline{c} }_{b-N}({{\varvec{r}}}_{cube})$ are presented in Subsection 3.3 of SI.

**Figure 4:**
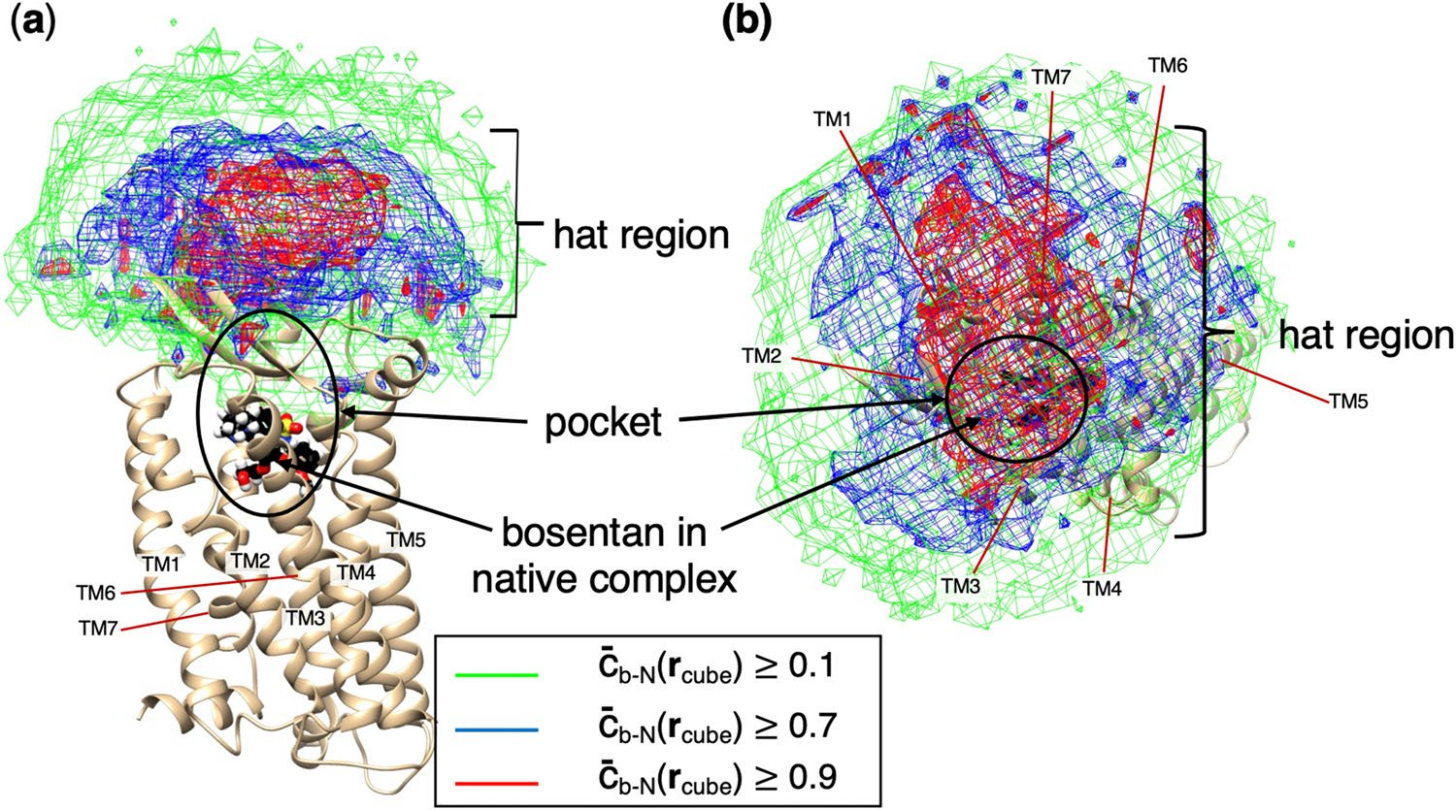
(**a**) Spatial patterns of cube-based bosentan–tail contact ratio $\overline{c}_{b - N} \left( {{\varvec{r}}_{cube} } \right)$ at three contour levels presented in the inset. The “hat” region, “pocket”, and “bosentan in native complex” are also shown. (**b**) Spatial patterns viewed from above the pocket. The structure which is presented is the native complex. Transmembrane helices (TM1–7) are labeled.

Figure 4a presents the spatial patterns of ${\overline{c} }_{b-N}\left({{\varvec{r}}}_{cube}\right)$ at three contour levels: ${\overline{c} }_{b-N}\left({{\varvec{r}}}_{cube}\right)\ge 0.1$, $0.7$, and $0.9$. The low-contact contours, ${\overline{c} }_{b-N}\ge 0.1$, extended to regions distant from the gate of the binding pocket, mean that the N-terminal tail contacted bosentan even when bosentan was floating in solution. With bosentan approaching the pocket, the contact ratio increased quickly and reached ${\overline{c} }_{b-N}\ge 0.7$. Because the region of ${\overline{c} }_{b-N}\ge 0.7$ involved the density-hat region (${\rho }_{CMb}\ge 0.01$; magenta-colored contour regions of Fig. 2), the contact might act as an attractive interaction. The high-contact region of ${\overline{c} }_{b-N}\ge 0.9$ (magenta-colored contours in Fig. 4a) was located immediately above the pocket, not in the hETB binding pocket. It is particularly interesting that ${\overline{c} }_{b-N}\left({{\varvec{r}}}_{cube}\right)$ was considerably small in the pocket. The high contact between the tail and bosentan right outside the pocket, followed by loss of contact once bosentan is in the pocket, indicates that the role of the N-terminal tail is the recruiting of bosentan into the pocket.

It is worth recalling that the gate-closing occurs because of the contact of the N-terminal tail and the $\beta$-hairpin (Figure S7b). In other words, gate-opening is induced by the N-terminal tail fluctuating in solution away from the gate. Figure 4 shows that the regions of ${\overline{c} }_{b-N}\ge 0.7$ are widely extended far from the binding pocket. The gate-opening and closing, the large fluctuations of the N-terminal tail, and the bosentan–tail contact formation are mutually related.

### Bosentan slides along the N-terminal tail upon binding

As shown in Fig. 4, the cube-based bosentan–tail contact ratio depends on the relative position of bosentan to hETB in the 3D real space. Here, we quantify the bosentan position relative to hETB by quantity ${r}_{bb}$, which is the distance between the bosentan centroid, ${{\varvec{r}}}_{CMb,i}$, in snapshot $i$ and that in the native complex ${{\varvec{r}}}_{CMb}^{ref}$: ${r}_{bb}=|{{\varvec{r}}}_{CMb,i}-{{\varvec{r}}}_{CMb}^{ref}|$. Figure S7c presents ${r}_{bb}$ in a snapshot. Smaller ${r}_{bb}$ is associated with closer bosentan to its natively binding position. Then, we divided the ${r}_{bb}$ axis into 13 slices with thickness of $5$ Å, which are designated respectively as ${\Delta }_{k}$ ($k=1,\dots ,13$): The lower and upper boundaries of ${\Delta }_{k}$ are given respectively as $5(k-1)$ and $5k$ (unit is Å). Table S3 presents the slice range exactly. As explained in Section 7 of SI and in Figure S13, the boundary between slices ${\Delta }_{4}$ and ${\Delta }_{5}$ coincides well with the gate of the binding pocket, which was introduced as the boundary between the blue contour region (${\rho }_{CMb}({{\varvec{r}}}_{cube})\ge 0.1$) and the magenta-colored contour region (${\rho }_{CMb}({{\varvec{r}}}_{cube})\ge 0.01$) in Fig. 2. By this arrangement, four slices ${\Delta }_{1}$,…, ${\Delta }_{4}$ are in the binding pocket, and slices ${\Delta }_{5}$,…, ${\Delta }_{13}$ are outside the pocket.

Figures S14–S17 present conformations selected from some slices. Section 8 of SI explains some features of those selected conformations. These figures visually imply that the contact site on the N-terminal tail to bosentan tends to move with ${r}_{bb}$ motion. To verify this tendency quantitatively, we introduced a “residue-based bosentan–tail contact ratio” ${\rho }_{cnt}^{{\Delta }_{k}}\left(j\right)$, which is the contact ratio of residue $j$ of the N-terminal tail to bosentan in a slice ${\Delta }_{k}$. Then, we converted ${\rho }_{cnt}^{{\Delta }_{k}}\left(j\right)$ to a potential of mean force ($PMF$) ${F}^{{\Delta }_{k}}\left(j\right)$ (equation S14 of Section 9 of SI). One can imagine a 2D plane constructed by the residue ordinal number $j$ and the slice ${\Delta }_{k}$. The lower the ${F}^{{\Delta }_{k}}\left(j\right)$ at a site $[j,{\Delta }_{k}]$ in the 2D plane, the stabler the contact becomes.

**Figure 5:**
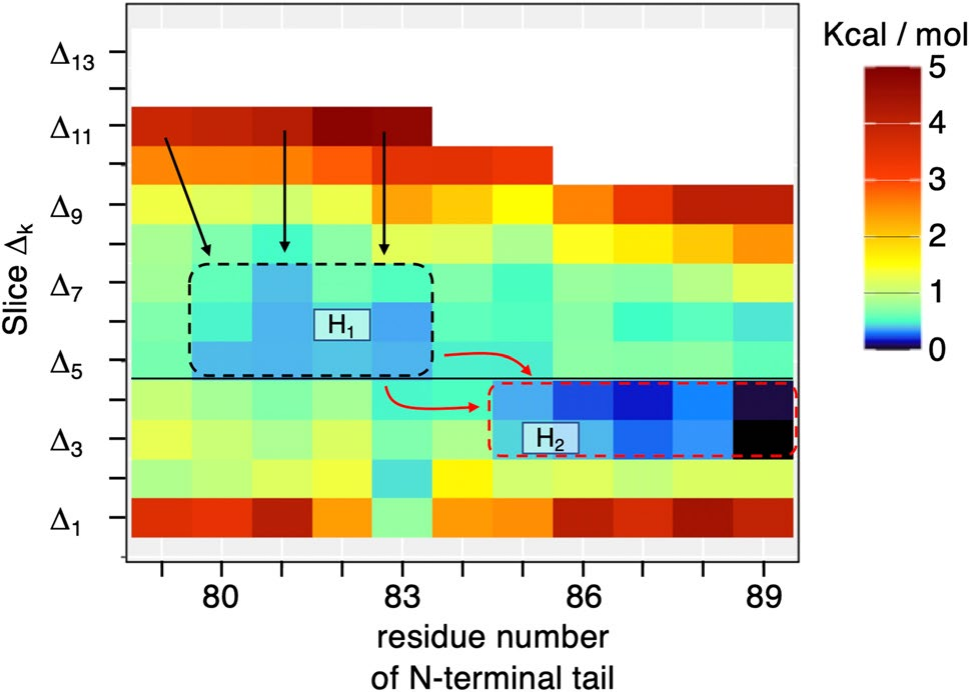
Residue-based bosentan–tail contact ratio, $\rho_{cnt}^{{{\Delta }_{k} }} \left( j \right)$, converted to $PMF$: $F^{{{\Delta }_{k} }} \left( j \right) = - RTln\left[ {\rho_{cnt}^{{{\Delta }_{k} }} \left( j \right)} \right]$ (see Section 9 of SI), where $j$ is the residue ordinal number (x-axis) of hETB’s N-terminal tail, and where ${\Delta }_{k}$ is the $k$-th $r_{bb}$ slice (y-axis), where the respective lower and upper boundaries are $5\left( {k - 1} \right)$ and $5k$. Unit is Å. $PMF$ value is presented by color (see color bar). White is assigned to unsampled regions or regions for which the $PMF$ is larger than 5 kcal/mol. The sequence of the N-terminal tail is Ace, Ser80, Pro81, Pro82, Arg83, Thr84, Ile85, Ser86, Pro87, Pro88, Pro89, where Ace is an acetyl group introduced to cap the N-terminal tail and where the residue number of 79 is assigned to Ace in the X-axis of the figure. Also, ${\Delta }_{k}$ is the range of the $r_{bb}$ slice given in Table S3. Region $H_{1}$ ranges from Ser80 to Arg83 in x-axis and from ${\Delta }_{7}$ to ${\Delta }_{5}$ in y-axis. $H_{2}$ does from Ile85 to Pro89 and from ${\Delta }_{4}$ to ${\Delta }_{3}$. The meanings of arrows are described in the text. The gate of the binding pocket is defined by boundary (thin line) between ${\Delta }_{4}$ and ${\Delta }_{5}$ and the binding pocket is the region below the gate (${\Delta }_{4}$, ${\Delta }_{3}$, ${\Delta }_{2}$, and ${\Delta }_{1}$).

Figure 5 shows ${F}^{{\Delta }_{k}}\left(j\right)$ in the 2D plane as the free-energy landscape. This figure indicates the existence of two free-energy basins, which are denoted as ${H}_{1}$ and ${H}_{2}$ in the figure. We discuss the binding process assuming that bosentan is approaching hETB from a far solvent region to the binding pocket. In ${\Delta }_{13}$ (60  Å $\le {r}_{bb}<65$ Å), the N-terminal tail does not touch bosentan: ${\rho }_{cnt}^{{\Delta }_{13}}\left(j\right)=0$ ($\forall j$). In ${\Delta }_{12}$ (55 Å $\le {r}_{bb}<60$ Å), bosentan first touches Ace (N-terminal capping group) of the N-terminal tail. Because the system moves generally toward a low free-energy region, possible transition paths are indicated by black arrows in Fig. 5, which lead the conformation to basin ${H}_{1}$. Those motions decrease ${r}_{bb}$ and the bosentan–tail contact firm, thereby decreasing the free energy.

With further decrease of ${\Delta }_{k}$, the stable region moved from the basin ${H}_{1}$ to ${H}_{2}$, i.e., the system moves from the tip region to the root region of the N-terminal tail. This move is natural because basin ${H}_{2}$ is lower than basin ${H}_{1}$. One can recall that the boundary between ${\Delta }_{5}$ and ${\Delta }_{4}$ corresponds to the gate of binding pocket. Conformational changes from ${H}_{1}$ to ${H}_{2}$ can occur via multiple pathways indicated by the red arrows in Fig. 5. Because the sites to which the red arrows are assigned had a larger free energy than either ${H}_{1}$ or ${H}_{2}$, the ${H}_{1}$-${H}_{2}$ basin switch requires that the free-energy barrier be overcome.

In ${\Delta }_{2}$ and ${\Delta }_{1}$, which are located at the bottom of the binding pocket, the bosentan–tail contact is disrupted. This disruption does not mean that the complex itself is dissociated but that the complex is stabilized without the bosentan–tail contact because the native-like complex conformations are stabler than the other state, as shown in Figs. 2 and 3. We also observed a specific contact at Arg83 in ${\Delta }_{2}$ and ${\Delta }_{1}$. Nevertheless, this specific contact represents a weak interaction because the depth of free energy at Arg83 is shallow (Fig. 5).

The importance of the long N-terminal tail is demonstrated by comparing the current simulation with the simulations from GPCRmd^46^, which is a database of various MD simulations of GPCRs (https://submission.gpcrmd.org/home/). In the unbound-form MD simulation of hETB in GPCRmd (https://submission.gpcrmd.org/view/886), the N-terminal tail of hETB fluctuates greatly in solvent without forming a specific conformation. This result agrees with those obtained from our study. By contrast, in the bound-form simulation of hETB from GPCRmd (https://submission.gpcrmd.org/view/887), the N-terminal tail contacted frequently to a ligand, which bound in the deep binding pocket of hETB. Figure 5 shows that the bosentan-tail contact disappeared when bosentan moved to the bottom of the binding pocket in our simulation. This discrepancy of the tail conformation in the bound form between our simulation and the simulation from GPCRmd is caused simply by a difference in the length of the tail used for the two simulations. The N-terminal tail in the current simulation is six residues longer than that of the GPCRmd simulation. The longer N-terminal tail cannot be packed into the binding pocket of GPCR. We presume that our simulation is more realistic than the bound-form simulation of GPCRmd because GPCRs have a long N-terminal tail in general. Therefore, this comparison of the two simulations demonstrates the importance of the length of the N-terminal tail.

### Bosentan–tail non-specific contacts

**Figure 6:**
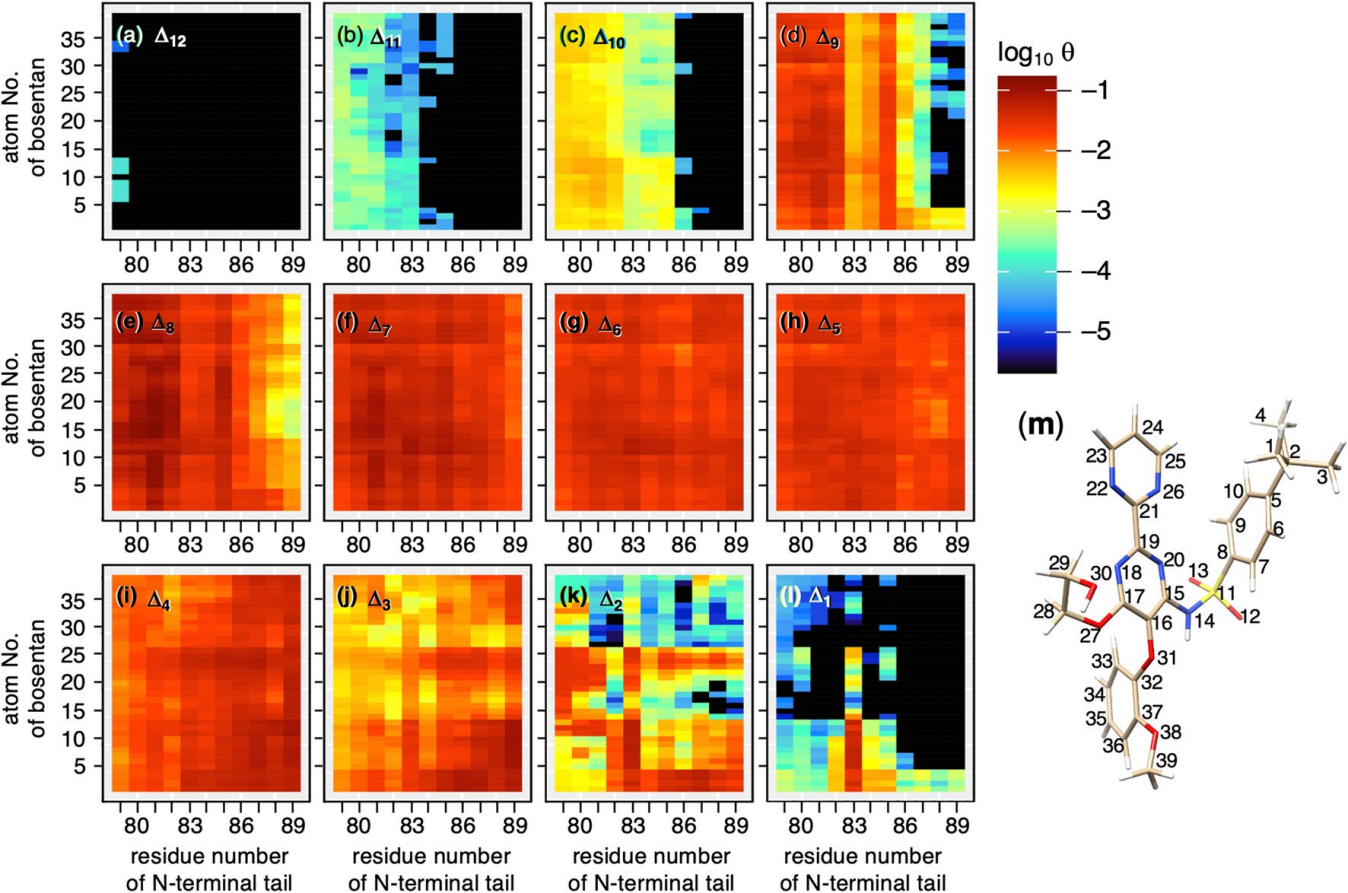
Atom–residue contact ratio $\theta_{cnt}^{{{\Delta }_{k} }} \left( {i,j} \right)$, which is the contact ratio of heavy atom $i$ of bosentan to residue $j$ of N-terminal tail in each slice ${\Delta }_{k}$. Its exact definition is given in equation S15 of SI. Panels (**a**–**l**) respectively show $\theta_{cnt}^{{{\Delta }_{12} }} \left( {i,j} \right), \cdots , \theta_{cnt}^{{{\Delta }_{1} }} \left( {i,j} \right)$. (**m**) Atom ordinal numbers assigned to bosentan's heavy atoms.

Figure 5 does not show whether the bosentan–tail contact is specific or non-specific. To investigate the bosentan atom specificity, we calculated an “atom–residue contact ratio” ${\theta }_{cnt}^{{\Delta }_{k}}\left(i,j\right)$ which is the contact ratio of a heavy atom $i$ of bosentan contacting residue $j$ of the N-terminal tail in each slice ${\Delta }_{k}$. The exact definition of ${\theta }_{cnt}^{{\Delta }_{k}}\left(i,j\right)$ is presented in Section 9 of SI. Figure 6 shows ${\theta }_{cnt}^{{\Delta }_{k}}\left(i,j\right)$ in each slice. Normalization is done for each panel of Fig. 6 to highlight the atom-specificity (color graduation) in each slice. Consequently, the existence of basins ${H}_{1}$ or ${H}_{2}$ is not recognized in Fig. 6.

Figure 6a shows that a few atoms of bosentan rarely contact Ace of the N-terminal tail in ${\Delta }_{12}$. Figures 6b–d depict that all the heavy atoms of bosentan contact non-specifically to the tip of the N-terminal tail in ${\Delta }_{11}-{\Delta }_{9}$. Figures 6e–h again show that bosentan contact is non-specific in ${\Delta }_{8}-{\Delta }_{5}$, where all residues of the N-terminal tail contact to bosentan. Up to ${\Delta }_{12}-{\Delta }_{5}$, bosentan is outside of the binding pocket. The non-specific contact is still found in ${\Delta }_{4}$ (Fig. 6i) and ${\Delta }_{3}$ (Fig. 6j), although ${\Delta }_{4}$ and ${\Delta }_{3}$ are in the binding pocket.

In ${\Delta }_{2}$ and ${\Delta }_{1}$ (Fig. 6k,l, respectively), by contrast, Arg83 of the N-terminal tail contacts specifically to atoms 1–13 of bosentan, as shown in Fig. 6m. This specificity is consistent with the depiction in Fig. 5. Atoms 1–10 and 11–13 are associated respectively with the hydrophobic and hydrophilic groups. Figure S18 presents some conformations that involve the specific bosentan–tail contacts. Generally, the hydrophobic sidechain stem of Arg83 of the tail and the hydrophobic group (atoms 1–10) of bosentan share some contact, as do the nitrogen atoms of the sidechain tip of Arg83 and oxygen atoms of sulfonamide of bosentan.

As shown in Figs. 4 and 5, the bosentan–tail contact tends to be dissociated when bosentan proceeds to the bottom of the binding pocket (${\Delta }_{1}$ and ${\Delta }_{2}$). Consequently, in many snapshots depicting ${\Delta }_{1}$ and ${\Delta }_{2}$, the N-terminal tail does not contact bosentan. If the bosentan–tail contact has a role to capture bosentan in solution, then we consider that the contact in the binding pocket has no role for molecular binding.

### Fly casting with ligand sliding

Here we check whether the bosentan–tail contact acts as an attractive interaction. First, we calculated a probability distribution function ${P}_{{\Delta }_{k}}({r}_{b-N})$ regarding the minimum heavy atomic distance ${r}_{b-N}$ from bosentan to the N-terminal tail for snapshots, which are involved in a slice ${\Delta }_{k}$. Then, $PMF$ regarding ${r}_{b-N}$ is defined as ${PMF}_{{\Delta }_{k}}\left({r}_{b-N}\right)=-RT\mathrm{ln}[{P}_{{\Delta }_{k}}({r}_{b-N})]$. In fact, the effective interaction between two molecules is contributed by many elemental interactions. To estimate this interaction comprehensively, $PMF$ is the best quantity. This quantity involves not only direct intermolecular interactions but also indirect interactions (ligand–solvent and solvent–receptor interactions). This quantity also involves effects of molecular motions (i.e., conformational variety, or entropy).

**Figure 7:**
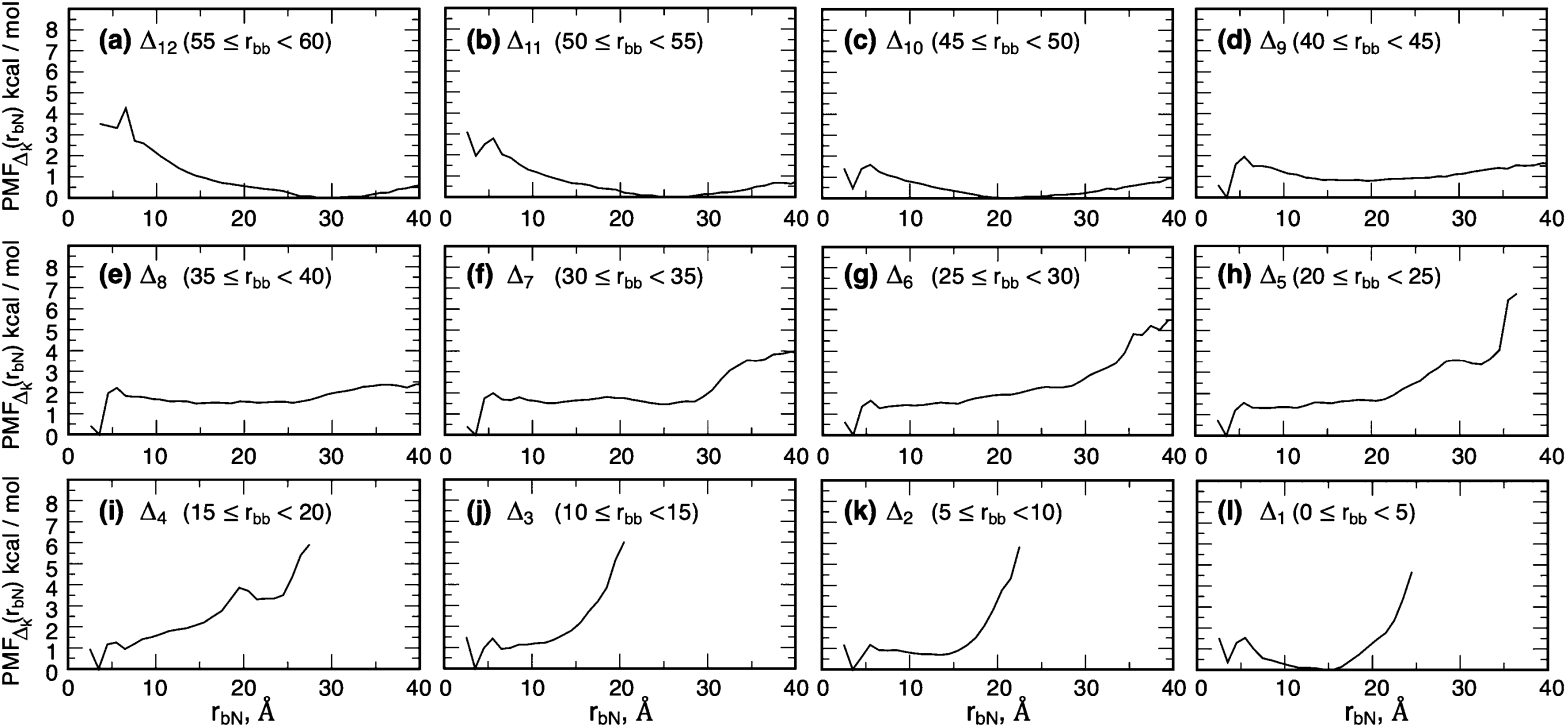
Potential of mean force $PMF_{{{\Delta }_{k} }} \left( {r_{b - N} } \right)$ for the bosentan–tail distance $r_{b - N}$ in slice ${\Delta }_{k}$ ($k = 1, \ldots 12$). $PMF_{{{\Delta }_{k} }} \left( {r_{b - N} } \right)$ is defined in the main text. Panels (**a**–**l**) respectively show $PMF_{{{\Delta }_{12} }} \left( {r_{b - N} } \right), \cdots ,PMF_{{{\Delta }_{1} }} \left( {r_{b - N} } \right)$. Also, $PMF_{{{\Delta }_{13} }} \left( {r_{b - N} } \right)$ is omitted because $PMF_{{{\Delta }_{13} }} \left( {r_{b - N} } \right)$ is similar with $PMF_{{{\Delta }_{12} }} \left( {r_{b - N} } \right)$.

Figure 7 presents $PMF$ for each slice. Apparently, the bottom of the lowest free-energy basin is assigned to a noncontact state in slices ${\Delta }_{12}$–${\Delta }_{10}$ (${r}_{bb}\ge 45$ Å) (Fig. 7a–c). Actually, $PMF$ for ${\Delta }_{13}$ is not shown because a bosentan–tail contact was not detected in ${\Delta }_{13}$. In contrast, the lowest free-energy basin is assigned to a contact state (${r}_{b-N}\approx 3.5$ Å) in ${\Delta }_{9}$–${\Delta }_{2}$ (Fig. 7d–k), which indicates that the bosentan–tail contact acts effectively as an attractive interaction in the range of 45 Å $>{r}_{bb}\ge$ 5 Å.

The analysis described above showed that a bosentan–tail attractive interaction exerted from ${\Delta }_{9}$ (45 Å $={r}_{bb}$) between the N-terminal tail and bosentan. Together with the residue sliding from the tip side to the root side with decreasing ${r}_{bb}$, we designate this binding mechanism “Fly Casting with ligand sliding”.

### Bosentan–membrane contact

Bosentan occasionally contacts the membrane (Figures S14e, S15d and S15e). To investigate the bosentan–membrane contact, we introduced a quantity, a “bosentan–membrane contact ratio” ${\overline{c} }_{b-m}\left({{\varvec{r}}}_{cube}\right)$, that is calculated with a similar procedure with ${\overline{c} }_{b-N}\left({{\varvec{r}}}_{cube}\right)$. Subsection 3.3 of SI provides details of the calculation procedure. The bosentan–membrane contact was formed commonly on the membrane surface (Figure S19). Some GPCR ligands are known to diffuse from the lipid bilayer to reach the binding pocket of GPCRs rather than approaching the pocket from the solvent side^47,48^. From the present simulation, however, we were unable to find such a ligand-diffusive motion of bosentan in the membrane, whereas we observed that bosentan sank in the membrane (Figure S14e). One might consider that the absence of bosentan diffusive motions in membrane might be due to insufficient setting of the upper boundaries of ${\lambda }^{(\beta )}$ and ${\lambda }^{(\gamma )}$ (68.47 Å and 67.47 Å, respectively; Table S2). However, Figure S11 of SI shows that the upper boundaries were sufficiently large for bosentan to move in the membrane if no interaction is applied between bosentan and the membrane. Therefore, we conclude that bosentan mainly approaches the pocket from solvent. Because the membrane prevents bosentan from moving to the cytoplasmic side, the membrane has a role in restricting the phase volume for bosentan to move. This volume restriction might facilitate binding of bosentan to the receptor’s binding pocket^49^.

### Orientational selection when bosentan enters the binding pocket

We investigated the molecular orientation of bosentan around hETB. First, we defined the bosentan molecular orientation by two vectors (orientation vectors) ${{\varvec{v}}}_{\downarrow }$ and ${{\varvec{v}}}_{\leftarrow }$, which are fixed in the core region of bosentan (Figure S20a), which are approximately perpendicular to one another. Figure 3a gives the exact definition of the core region. Then, we normalized the orientation vectors as ${|{\varvec{v}}}_{\alpha }|=1$ ($\alpha =\leftarrow \mathrm{or} \downarrow$). Because the bosentan core region is structurally stiff, the two orientation vectors are convenient quantities for defining the bosentan molecular orientation. In fact, in general, two nonparallel vectors can specify the orientation of a plane (the central ring of the core region) in 3D real space. Figure S20b presents an example of the vectors in the native complex and in a snapshot. Next, we assigned the orientation vectors to a cube ${{\varvec{r}}}_{cube}$, in which the bosentan centroid was detected. Then, Ω in equation S7 of Subsection 3.4 of SI is replaced by ${{\varvec{v}}}_{\alpha }$. The resultant quantity $\overline{\Omega }\left({{\varvec{r}}}_{cube}\right)$ is designated as ${\overline{{\varvec{v}}} }_{\alpha }\left({{\varvec{r}}}_{cube}\right)$ in the equation. We designated ${\overline{{\varvec{v}}} }_{\alpha }\left({{\varvec{r}}}_{cube}\right)$ as an “averaged orientation vector”. Because ${{\varvec{v}}}_{\alpha }$ in a snapshot was normalized, the maximum of $\left|{\overline{{\varvec{v}}} }_{\alpha }\right|$ is unity when all the vectors detected in the cube ${{\varvec{r}}}_{cube}$ are perfectly ordered. The minimum is 0.0 when the vectors are randomized completely.

**Figure 8:**
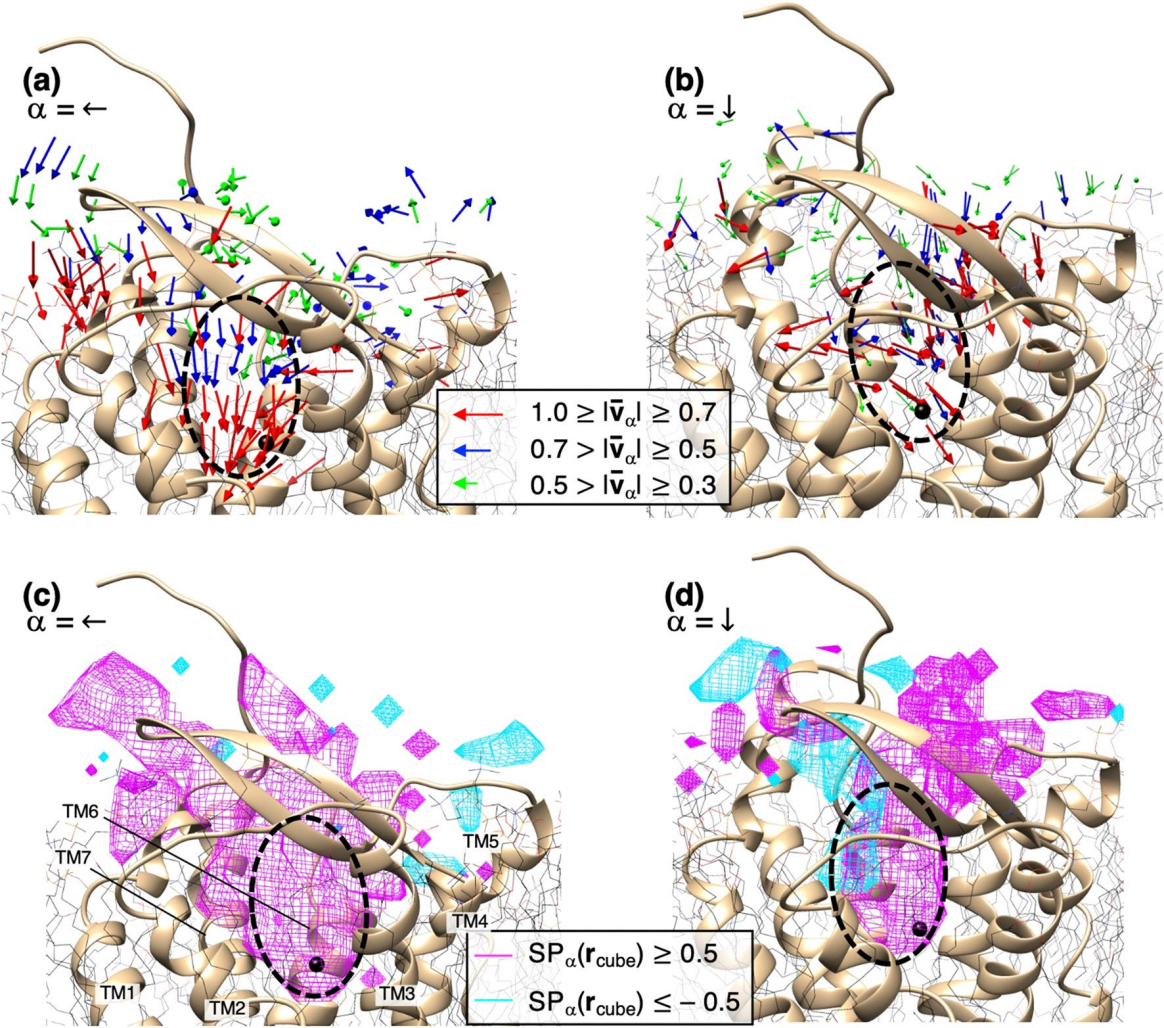
Spatial patterns of averaged orientation vectors (**a**) $\overline{\user2{v}}_{ \leftarrow } \left( {{\varvec{r}}_{cube} } \right)$ and (**b**) $\overline{\user2{v}}_{ \downarrow } \left( {{\varvec{r}}_{cube} } \right)$. A vector is colored differently by its norm $\left| {\overline{\user2{v}}_{\alpha } \left( {{\varvec{r}}_{cube} } \right)} \right|$ ($\alpha = \leftarrow$ or $\alpha = \downarrow$) (inset). Vectors with $\left| {\overline{\user2{v}}_{\alpha } \left( {{\varvec{r}}_{cube} } \right)} \right| < 0.3$ are omitted. (**c**) Spatial patterns of scalar product $SP_{ \leftarrow } \left( {{\varvec{r}}_{cube} } \right)$ (= ${\varvec{e}}_{{\overline{\user2{v}}_{ \leftarrow } }} \left( {{\varvec{r}}_{cube} } \right)\;\cdot{\varvec{v}}_{ \leftarrow }^{{\left( {ref} \right)}}$) and (**d**) those of $SP_{ \downarrow } \left( {{\varvec{r}}_{cube} } \right)$ (= ${\varvec{e}}_{{\overline{\user2{v}}_{ \downarrow } }} \left( {{\varvec{r}}_{cube} } \right)\;\cdot{\varvec{v}}_{ \downarrow }^{{\left( {ref} \right)}}$), where ${\varvec{e}}_{{\overline{\user2{v}}_{\alpha } }} \left( {{\varvec{r}}_{cube} } \right)$ and ${\varvec{v}}_{\alpha }^{{\left( {ref} \right)}}$ are defined in main text. Regions of $SP_{\alpha } \left( {{\varvec{r}}_{cube} } \right) \ge 0.5$ and $SP_{\alpha } \left( {{\varvec{r}}_{cube} } \right) \le - 0.5$ are colored differently by their values (inset). For all panels, the hETB structure is from the native complex. Small black spheres are bosentan centroid in the native complex. The binding pocket is shown as a broken-line circle in all panels. Transmembrane helices (TM1–7) are labeled for panel c. Positions of the transmembrane helices for the other panels are presumable from panel c because hETB is shown from similar directions for all panels.

Figures 8a,b portray spatial patterns of ${\overline{{\varvec{v}}} }_{\alpha }\left({{\varvec{r}}}_{cube}\right)$, in which orientation vectors with large norms ($\left|{\overline{{\varvec{v}}} }_{\alpha }\right|>0.3$) are shown. Importantly, the large-norm vectors were found mainly in the binding pocket. Therefore, the bosentan orientation is ordered in the pocket, although it is disordered outside the pocket. This finding suggests that configurational entropy of bosentan decreases quickly when bosentan passes the gate of the binding pocket. It is worth recalling that the free-energy basin of ${F}^{{\Delta }_{k}}\left(j\right)$ also switched quickly from ${H}_{1}$ to ${H}_{2}$ at the gate (Fig. 5). In fact, the decrease of entropy results in increased free energy. This entropic decrease should be compensated by another thermodynamic factor. Otherwise, bosentan in the binding pocket is destabilized, and the density ${\rho }_{CMb}({{\varvec{r}}}_{cube})$ in the binding pocket decreases. We show in the next section that intermolecular native contacts, which act as attractive interactions between bosentan and hETB, are formed in the binding pocket. Consequently, those intermolecular attractive interactions (enthalpy decrease) compensate the entropy loss.

However, Fig. 8a,b do not necessarily mean that the bosentan orientations resemble those in the native-complex position. Then, we check the orientation similarity as follows: First, we calculated ${{\varvec{v}}}_{\leftarrow }$ and ${{\varvec{v}}}_{\downarrow }$ for the native complex, which are designated respectively as ${{\varvec{v}}}_{\leftarrow }^{(ref)}$ or ${{\varvec{v}}}_{\downarrow }^{(ref)}$. Next, we calculated a scalar product as $S{P}_{\alpha }\left({{\varvec{r}}}_{cube}\right)={{\varvec{e}}}_{{\overline{{\varvec{v}}} }_{\boldsymbol{\alpha }}}\left({{\varvec{r}}}_{cube}\right)\cdot {{\varvec{v}}}_{\alpha }^{(ref)}$, where ${{\varvec{e}}}_{{\overline{{\varvec{v}}} }_{\boldsymbol{\alpha }}}\left({{\varvec{r}}}_{cube}\right)$ is a unit vector that is parallel to ${\overline{{\varvec{v}}} }_{\boldsymbol{\alpha }}\left({{\varvec{r}}}_{cube}\right)$. If ${{\varvec{e}}}_{{\overline{{\varvec{v}}} }_{\boldsymbol{\alpha }}}\left({{\varvec{r}}}_{cube}\right)$ is exactly parallel to ${{\varvec{v}}}_{\alpha }^{(ref)}$, then $S{P}_{\alpha }\left({{\varvec{r}}}_{cube}\right)=1$; if ${\overline{{\varvec{v}}} }_{\alpha }\left({{\varvec{r}}}_{cube}\right)$ is exactly anti-parallel to ${{\varvec{v}}}_{\alpha }^{(ref)}$, then $S{P}_{\alpha }\left({{\varvec{r}}}_{cube}\right)=-1$. If ${{\varvec{e}}}_{{\overline{{\varvec{v}}} }_{\boldsymbol{\alpha }}}\left({{\varvec{r}}}_{cube}\right)$ and ${{\varvec{v}}}_{\alpha }^{(ref)}$ are mutually perpendicular, then $S{P}_{\alpha }\left({{\varvec{r}}}_{cube}\right)=0$. Therefore, the spatial pattern of $S{P}_{\alpha }\left({{\varvec{r}}}_{cube}\right)$ is a useful measure to assess similarity between ${\overline{{\varvec{v}}} }_{\alpha }\left({{\varvec{r}}}_{cube}\right)$ and ${{\varvec{v}}}_{\alpha }^{(ref)}$.

Figure 8c demonstrates that the major volume of the binding pocket was occupied by $S{P}_{\leftarrow }\left({{\varvec{r}}}_{cube}\right)\ge 0.5$, which indicates similar orientations of ${\overline{{\varvec{v}}} }_{\leftarrow }$ and ${{\varvec{v}}}_{\leftarrow }^{(ref)}$. For ${\overline{{\varvec{v}}} }_{\downarrow }$, a large volume of the pocket was also characterized by $S{P}_{\downarrow }\left({{\varvec{r}}}_{cube}\right)\ge 0.5$ , whereas a minor part (cyan-colored contours) of the pocket was characterized by $S{P}_{\downarrow }\left({{\varvec{r}}}_{cube}\right)\le -0.5$ (Fig. 8d). This minor part does not cover the bosentan native position at all. Therefore, we can infer that bosentan enters the pocket with limited orientations that are beneficial for reaching the native-complex position because the pocket is too narrow for bulky bosentan to flip. We designate this possible mechanism as “orientational selection”.

### Bosentan–hETB contacts and ring-C packing at the bottom of binding pocket

In the above section, we suggested that the entropy loss (decrease in the orientiational variety of bosentan) should be compensated by enthalpy decrease for the stabilization of bosentan in the binding pocket. To investigate this stabilization effect, we analyze attractive interactions (atomic contacts) of bosentan with amino-acid residues of hETB in the binding pocket.

First, we observe common intermolecular interactions in the three X-ray structures: the bosentan–hETB^10^, ET1–hETB^6^, and the K8794–hETB^10^ complexes. In fact, the same binding scheme can be observed in the three complex structures (Figure S21). By viewing the three complexes closely, we identified four intermolecular native atomic contacts (interatomic distance less than 5 Å in the X-ray structures), which exist commonly in the three complex structures as attractive interactions, as shown in Section 10 of SI, Figures S22a, S22b, and Table S4: contact (1) between oxygen atoms in the sulfonyl group ($\mathrm{SOO}$) of the bosentan’s sulfonamide and the nitrogen (${N}_{\zeta }$) of Lys 182 (3 × 33); (2) between the oxygens in bosentan’s $\mathrm{SOO}$ and the nitrogen of Lys 273 (5 × 39); (3) between the oxygens in bosentan’s $\mathrm{SOO}$ and nitrogen atoms of Arg 343 (6 × 55); and (4) between the nitrogen atom ($N14$) of the bosentan’s sulfonamide and the nitrogen (${N}_{\zeta }$) of Lys 182 (3 × 33). The actual interatomic distances for the four contacts are listed in Table S4 for the three X-ray structures: Most of contacts are around 3 Å, with the sole exception of 4.56 Å for (2) in the bosentan–hETB complex. Figure S22c enumerates all intermolecular interactions in the bosentan–hETB complex.

Contacts (1)–(3) are associated with salt bridges. Contact (4) can be associated either with a salt bridge or with a hydrogen bond because we cannot infer whether a hydrogen atom is bound to $N14$ of the bosentan’s sulfonamide in the X-ray crystallography, or not. Nevertheless, it is noteworthy that both the salt bridge and the hydrogen bond are attractive interactions. For the present study, we used the neutral form for bosentan, in which a hydrogen is attached to $N14$ (Fig. 1a). This ambiguity of protonation is discussed at the end of the “Results and Discussion” section.

The native atomic contacts introduced above are appropriate quantities to analyze the stabilization effect (enthalpic effect) that compensates the entropic disadvantage (decrease of molecular orientation). As more native contacts form in the complex, the attractive interactions become stronger while bosentan’s structural entropy decreases.

To analyze the intermolecular distances of snapshots, we introduce the notation of ${r}_{\alpha ;\beta }$, which is a distance between atoms $\alpha$ and $\beta$ belonging respectively to the ligand (bosentan, K8794, or ET1) and hETB. We use the original PDB residue numbering to specify atom $\beta$. The four distances related to the four native contacts are designated as ${r}_{\mathrm{N}14;{\mathrm{Lys}182\mathrm{N}}_{\upzeta }}$, ${r}_{\mathrm{SOO};{\mathrm{Lys}182\mathrm{N}}_{\upzeta }}$, ${r}_{\mathrm{SOO};{\mathrm{Lys}273\mathrm{N}}_{\upzeta }}$, and ${r}_{\mathrm{SOO};{\mathrm{Arg}343\mathrm{N}}_{\upeta }}$. When multiple distances were possible in ${r}_{\alpha ;\beta }$, the minimum of the distances was selected for ${r}_{\alpha ;\beta }$. For instance, four distances are possible for ${r}_{\mathrm{SOO};{\mathrm{Arg}343\mathrm{N}}_{\upeta }}$ because two oxygen atoms and two nitrogen atoms are involved respectively in the sulfonyl group and Arg 343 (6 × 55). Also, Table S4 presents distances ${r}_{\alpha ;\beta }$ in the X-ray structures, in which three distances are approximately 3 Å, except for $r_{\text{SOO}};{\text{Lys}}182{\text{N}}{\zeta }$ (= 4.56 Å) from the bosentan-hETB complex. We presume that ${r_{\text{SOO}};{\text{Lys}}182{\text{N}}\zeta}$, $r_{{{\text{SOO}};{\text{Lys}}273{\text{N}{\zeta }}}}$, and $r_{{{\text{SOO}};{\text{Arg}}343{\text{N}{\eta }}}}$ are related to salt-bridges, and that $r_{{{\text{N}}14;{\text{Lys}}182{\text{N}{\zeta }}}}$ might be done to either a salt-bridge or a hydrogen bond.

We judged that the heavy atoms $\alpha$ and $\beta$ are contacting if $r_{\alpha ;\beta } < 5.0$ Å is satisfied in a snapshot. The value of 5.0 Å is about $0.5$ Å larger than the largest $4.56$ Å) of the experimentally obtained values (Table S4). Considering thermal fluctuations of the complex structure and the likelihood of error in experiment measurements (3.6 Å resolution for the bosentan–hETB complex), we added a tolerance figure of $0.5$ Å to the inequality. Furthermore, not only does a heavy atom have a radius of approximately $2.0$ Å, but the remaining distance between $\alpha$ and $\beta$ is $1.0$ Å (= 5.0  Å − 2 × 2.0 Å), which does not allow a water molecule to penetrate because the water molecule diameter is about 3.0 Å. Therefore, the $5.0$ Å threshold is rational to check the atomic contacts.

Based on the criteria above, we calculated the number of contacts $N_{cnt}$ ($0 \le N_{cnt} \le 4$) regarding the four distances for all snapshots. Then, picking snapshots falling in an $r_{bb}$-range of $R\left( {r_{bb} } \right) = \left[ {r_{bb} - \Delta r_{bb} ; r_{bb} + \Delta r_{bb} } \right]$, we calculated the average of $N_{cnt}$ for the selected snapshots and denoted it as $N_{cnt} \left( {r_{bb} } \right)$. Section 11 of SI presents the exact method to calculate $N_{cnt} \left( {r_{bb} } \right)$ and its standard deviation $SD_{cnt} \left( {r_{bb} } \right)$. Ideally, $\Delta r_{bb}$ should be sufficiently small ($\Delta r_{bb} \to 0$) to calculate $N_{cnt} \left( {r_{bb} } \right)$ exactly at $r_{bb}$. However, the number of snapshots in $R\left( {r_{bb} } \right)$ decreases concomitantly with decreasing $\Delta r_{bb}$ because the simulation length is finite. To maintain statistical significance in $N_{cnt} \left( {r_{bb} } \right)$, we set $\Delta r_{bb} = 0.25$ Å for the present simulation. The value of $N_{cnt} \left( {r_{bb} } \right)$ is four if all snapshots in $R\left( {r_{bb} } \right)$ always have four contacts and zero if no snapshot has any contact.

**Figure 9:**
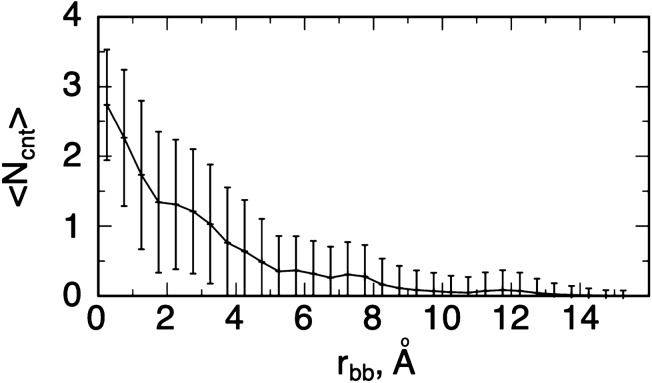
Dependence of $N_{cnt} \left( {r_{bb} } \right)$ on $r_{bb}$ and its standard deviation (error bars). Lower limits of some error bars are smaller than 0 on the x-axis because the standard deviation was calculated using the usual form: $\left[ {N_{cnt} \left( {r_{bb} } \right)^{2} - N_{cnt} \left( {r_{bb} } \right)^{2} } \right]^{1/2}$.

Figure 9 depicts the $r_{bb}$ dependence of $N_{cnt} \left( {r_{bb} } \right)$, where $r_{bb}$ starts from $0.25$ Å and where $N_{cnt}$ (0.25 Å) is calculated for the range of *R*(0.25 Å) = [0.0 Å; 0.5 Å]. Note that$R( {r_{bb}}$ < 0.25 Å) is impossible to be calculated. We set the maximum of $r_{bb}$ at $15.25$ Å, where the used range is *R*(15.25 Å) = [15.0 Å; 15.5 Å]. Also, *R*(15.25 Å) is involved in the slice ${\Delta }_{4}$, immediately below the gate of the hETB’s binding pocket (Figure S13). Therefore, Fig. 9 shows that almost no atom contact is formed at the gate: *N*_cnt_ (15.25 Å) = 0.005. Also, $N_{cnt} \left( {r_{bb} } \right)$ increased concomitantly with decreasing $r_{bb}$ (with bosentan approaching the native-complex position): The number of contacts exceeded the value of 1 at $r_{bb} = 3.25$ Å: *N*_cnt_ (3.25 Å) $= 1.03 \pm 0.85$. Subsequently, the growth rate of $N_{cnt} \left( {r_{bb} } \right)$ rose, and $N_{cnt} \left( {r_{bb} } \right)$ reached the maximum at $r_{bb}$ = 0.25 Å: *N*_cnt_ (0.25 Å) $= 2.74 \pm 0.79.$ We further investigated formation of each contact, as described in Section 12 of SI. Figure S23 shows that each native contact is formed with decreasing $r_{bb}$.

Because the four intermolecular native contacts act as attractive interactions (Section 10 of SI), Fig. 9 shows that the complex structure is stabilized enthalpically by the native contacts when bosentan goes to the bottom of the binding pocket. We presume that the instability induced by the decrease of the entropy when bosentan passing the gate of the binding pocket is compensated by formation of the native contacts.

We checked the reproducibility of the packing of ring C to the bottom of the binding pocket (see Figure S21c for ring C of bosentan). For this purpose, we calculated the density map $\rho_{CMC} \left( {{\varvec{r}}_{cube} } \right)$ of the centroid of ring C using equation S3 of Subsection 3.1 of SI with replacement of the centroid of portion $X$ by the centroid of ring $C$. Figure S24 presents the high-density spot ($\rho_{CMC} \left( {{\varvec{r}}_{cube} } \right) \ge 0.8$) for ring C. Importantly, the high-density spot is localized in a region that overlaps with the ring-C position in the native complex. However, the contours of $\rho_{CMC} \left( {{\varvec{r}}_{cube} } \right) \ge 0.4$ show deviation from the native-complex position. We discuss this discrepancy between the computation and the native complex later.

### Bosentan conformations in the highest-density spot

As shown in Fig. 2, the bosentan centroid in the native complex structure was located on the fringe of the computed highest-density spot (red contours in Fig. 2). This section presents an explanation of bosentan conformations in the highest-density spot.

As stated earlier, the highest-peak position of $P_{RDF} \left( {RMSD_{whole}^{heavy} } \right)$ or $P_{DDF} \left( {RMSD_{whole}^{heavy} } \right)$ of Fig. 3 is larger than that of $P_{RDF} \left( {RMSD_{core}^{heavy} } \right)$ or $P_{DDF} \left( {RMSD_{core}^{heavy} } \right)$, which suggests that the sidechains of bosentan were somewhat disordered. To examine this disorder in greater detail, we collected snapshots from 3.5 Å $\le RMSD_{whole}^{heavy} \le$ 4.0 Å, which involve the highest peak of $P_{RDF} \left( {RMSD_{whole}^{heavy} } \right)$ (Fig. 3b).

**Figure 10:**
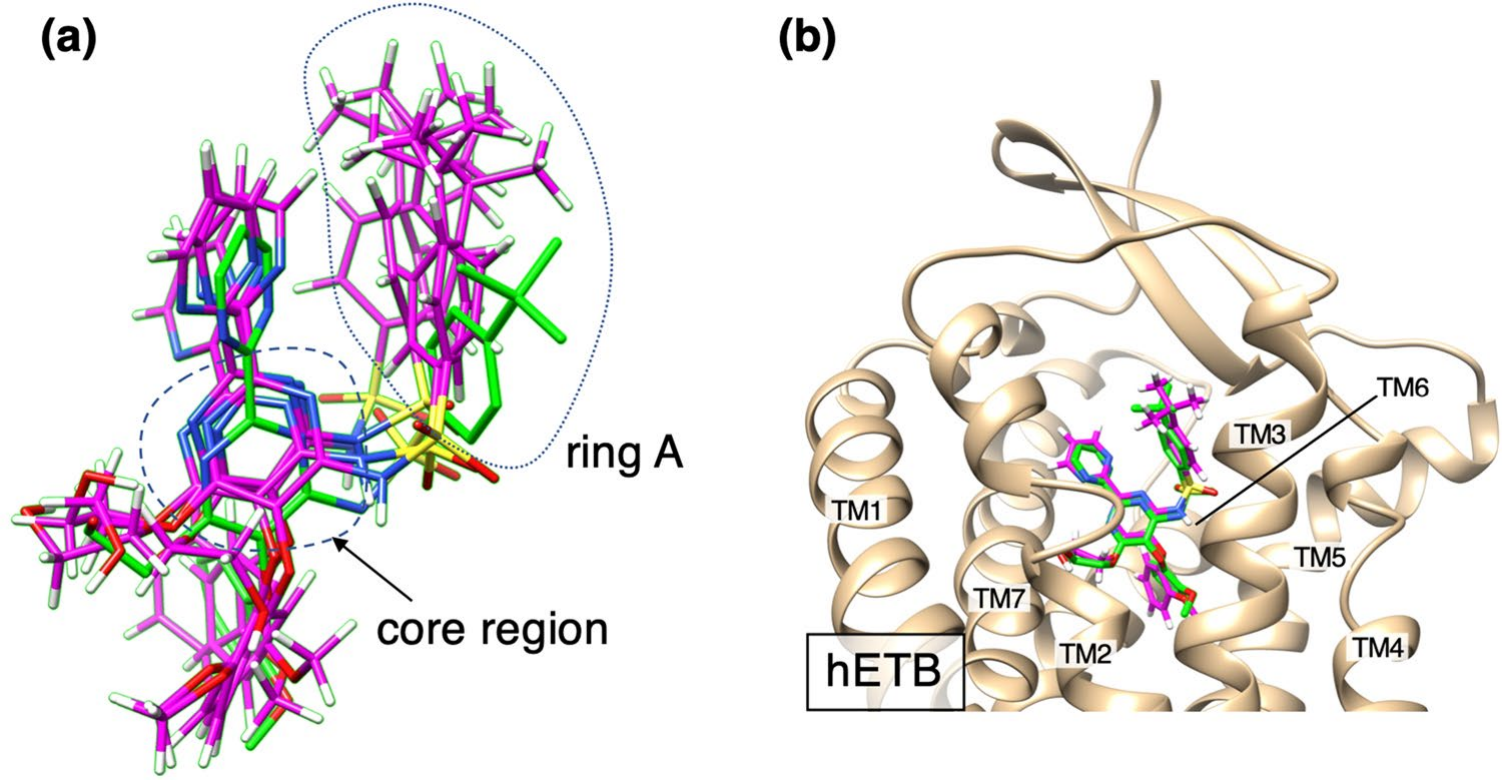
Snapshots sampled in the highest density spot ($\rho_{CMb} \left( {{\varvec{r}}_{cube} } \right) = 0.5$ in Fig. 3). In both panels, bosentan conformations from simulation are shown as magenta, and that in the native complex is shown as green. (**a**) Four conformations selected randomly from the range of 3.5 Å $\le RMSD_{whole}^{heavy} < 4.0$ Å. The bosentan core region and ring A are shown in the figure. (**b**) The conformation with $RMSD_{whole}^{heavy} = 1.11$ Å. hETB in the native complex is also shown. Although superimposition was not applied to bosentan, it was applied to trans-membrane helices of hETB (see Figure S7a). Transmembrane helices (TM1–7) are labeled for panel (**b**).

Figure 10a displays four randomly selected conformations from the snapshots collected above. This figure demonstrates that the conformations from the highest density spot have some similarity with the native-complex structure (the X-ray structure). Therefore, we referred to the complex conformations in highest density spot as “native-like complex”. However, Fig. 10a shows that the longest sidechain of bosentan, ring A (Figure S21c), is deviated from the native-complex position with a conformational diversity, whereas the core region was converged to a position close to the native-complex position. For this reason, the highest-peak position of $P_{RDF} \left( {RMSD_{whole}^{heavy} } \right)$ or $P_{DDF} \left( {RMSD_{whole}^{heavy} } \right)$ was larger than that of $P_{RDF} \left( {RMSD_{core}^{heavy} } \right)$ or $P_{DDF} \left( {RMSD_{core}^{heavy} } \right)$ in Fig. 3, respectively. We discuss this imperfection in the next section.

Figure 10b depicts a snapshot with a small $RMSD_{whole}^{heavy}$ ($1.11$ Å). Such small $RMSD_{whole}^{heavy}$ conformations were the minority in the ensemble of snapshots because there was no peak at $RMSD_{whole}^{heavy} \approx 1$ Å in Fig. 3. This small $RMSD_{core}^{heavy}$ was a result obtained because the ring A of this conformation overlapped well on that of the native-complex structure.

### Comparison of bosentan at the highest-density spot with that in the crystal complex structure

We note several discrepancies between bosentan in the crystal complex structure and that at the computed highest-density spot. Actually, GA-guided mD-VcMD is sufficiently powerful to explore the ligand–receptor conformational space starting from completely unbound conformations (Figure S5). However, we remark that reproduction of the native-like complex was not perfect. As discussed, the bosentan’s longest sidechain (ring A; Figure S21c) exhibited structural diversity in native-like complex conformations, although the core region agreed well with the native-complex position.

We infer two possibilities for the imperfectness: Accuracy of classical-mechanical force-fields and/or treatment of the bosentan’s sulfonamide. Despite multiple attempts to improve force fields, current force fields are well known to be imperfect. In future studies, novel force-field parameters might resolve details of conformational inconsistency with experimentation.

Regarding the second possibility, we used the bosentan’s sulfonamide in a normal (neutral) form in that a hydrogen atom is attached to the nitrogen atom (${\text{N}}14$) of the sulfonamide. In the neutral form, the electronic state of ${\text{N}}14$ is the ${\text{sp}}^{2}$ hybridization with planar three-chemical bonds (S-NH-C angle of approximately 130 degrees). In the deprotonated form, the electronic state is also the ${\text{sp}}^{2}$ hybridization with planar two-chemical bonds and one electron-lone pair (${\text{S}} = {{\ddot{N}}} - C$ angle of approximately 131°). Therefore, we cannot distinguish the electronic state of sulfonamide merely by viewing the X-ray structure of bosentan.

We might treat the ${\text{N}}14$ atom deprotonated because the $pKa$ value of bosentan is 5.1^50^ or 5.79^51^ in solution: 0.5% and 99.5% of bosentan molecules are in the neutral and ionic form, respectively, in human blood (${\text{pH}} = 7.4$). However, the form of sulfonamide in the hETB binding pocket is unknown. Reportedly^52^, the ${\text{pKa}}$ of sulfonamide in various compounds, which are immersed in a mixture of organic solvent and water, increases greatly with increasing fraction of the organic solvent. In addition, as reported^53^, the electronic state of amino acids in the protein interior might be different from that in solution. Furthermore, the cell membrane is negatively charged. The bosentan molecules in the negatively charged form (the major fraction in blood) should only slightly access the cell surface by the repulsive electrostatic force between the cell membrane and the negatively charged bosentan. In contrast, the neutral form (the minor fraction in blood) can reach the cell surface and the receptor. Finally, we decided to use the neutral form of bosentan with protonated ${\text{N}}14$.

As described earlier, an attractive interaction can act for either the neutral or charged form of bosentan to the nitrogen ($N_{\zeta }$) of Lys 182 (3 × 33). In fact, we demonstrated that the distribution function $\rho_{bs} ( {r_{N14;Lys182N\zeta } ;r_{bb} = 0.25}$ Å) exhibited a remarkable peak at $r_{N14;Lys182N\zeta } = 3$ Å (Figure S23f), which reproduces the experimentally obtained observations (Table S4). This result constitutes evidence that the currently used form of sulfonamide is plausible. To analyze the structure diversity of the ring A further, hugely numerous computations must be made in future studies, with varying of the force-fields and the charge state of sulfonamide.

### Binding mechanism from the current and other simulations

Recent advances in MD simulations have enabled investigation of ligand–GPCR interactions^54^. Various inter-molecular interactions and semi-stable complexes (poses) were investigated when the ligands reach the GPCR’s binding pocket^55^. Energetically favored binding pathways and stable states (poses) in a conformational space were investigated, and information transfer from the ligand binding pocket to the intracellular region of the receptor was analyzed from the obtained conformational ensemble^56–58^. A previous MD simulation study of ligands and the $\beta_{2}$-adrenergic receptor ($\beta_{2}$ AR) showed an energy barrier at the extracellular entrance of binding pocket^59^. The study suggested that the barrier is induced by dehydration of the ligand to enter the binding pocket. It is likely that this extracellular entrance may correspond to the “gate” of the binding pocket in our study because our simulation also showed the existence of a free-energy barrier, though this one is related to the ligand sliding on the hETB’s N-terminal tail and to the decrease of the ligand orientational variety (i.e., entropy loss) at the binding pocket gate. This difference of the origin for the free-energy barrier between these two studies implies the existence of a variety of GPCR binding pathways.

Most studies have focused on the ligand-binding pathways after the ligands have passed the gate of the GPCR’s binding pocket^56–59^. On the other hand, there has been little investigation where ligands being far from the binding pocket gate are concerned. One main outcome from the current study is that the disordered N-terminal tail acts as an attracter to capture the ligand. Therefore, these studies and ours complement each other other with respect to understanding different aspects of the ligand–GPCR binding process.

The mechanism through which N-terminal tail of a GPCR assists with ligand binding can also vary depending on the tail’s structure. For example, the N-terminal segment of a GPCR, sphingosine-1-phosphate receptor 1 (S1P_1_R), is not disordered but adapts an $\alpha$-helix in the crystal structure (PDB ID: 3v2y). This segment covers the gate of the binding pocket of the GPCR. A previous MD study^60^ reported that the ligand ML056 diffuses within the membrane to reach the entrance of the S1P_1_R's binding pocket. As the ligand passes the entrance, the $\alpha$-helix unfolds, increasing the width of the entrance, which allows the ligand to diffuse into the pocket more readily. This simulation study and ours demonstrate that there is variety in the ligand–GPCR binding process depending on the structure of the system being studied.

## Conclusions

Prediction of the ligand–receptor complex structure is crucially important for structure-based drug design. This strategy is reasonable to develop a new drug (especially antagonist) because the molecular system finally reaches the most thermodynamically stable structure irrespective of the binding pathways. However, the binding pathways are also important to elucidate the complex. As mentioned above, MD simulations showed ligand binding pathways of various GPCRs and semi-stable complex structures (poses) when the ligands enter into the binding pocket and reach the bottom of the binding pocket^5,7,54–56,58–60^. The current simulation study showed that an attractive interaction acts effectively between bosentan and the N-terminal tail of hETB via the fly casting mechanism^17–19^, even when bosentan is located distant from the binding pocket of hETB. Some experiments reported in the related literature have indicated that the N-terminal tails of GPCRs have important physiological functions^61^, whereas the N-terminal tails of GPCRs have been studied insufficiently in contrast to the binding pocket. Because the tail is conformationally disordered, a simulation like the one performed for this study is helpful for elucidating the functions or roles of the N-terminal tail in the binding process.

In general, GPCRs have a long and disordered N-terminal tail (40–200 amino-acid residues long)^21^. Results of an MD study revealed that an amino-acid substitution to the N-terminal tail of a GPCR (human $\beta_{2}$-adrenergic receptor) varies the tail dynamics, and that accessibility of the ligand to the binding pocket also varies by substitution^62^. This study, in addition to our present work, suggests that the ligand–receptor interaction should be studied even when the ligand is outside the binding pocket of GPCR.

The bosentan–hETB complex formation obtained from the present study is summarized as follows: First the tip-side of the N-terminal tail of hETB captures bosentan when bosentan is fluctuating in solution via nonspecific intermolecular contacts. The bosentan–capturing conformation is identified as the free-energy basin $H_{1}$ in the free-energy landscape (Fig. 5). Then ligand–sliding occurs occasionally from the tip-side to the root-side of the N-terminal tail when bosentan passes the gate of the binding pocket, which corresponds to basin switching from $H_{1}$ to the deeper basin $H_{2}$ with overcoming of the inter-basin free-energy barrier. We designated this mechanism as “fly-casting with ligand–sliding”. Importantly, the molecular orientational variety of bosentan decreases quickly when bosentan passes the gate (Fig. 8). This decrement of the orientational variety has a role of screening out bosentan conformations for which the molecular orientations are improper to reach the binding site (orientational selection). In fact, this orientational selection is a variant of conformational selection^20^. Because the decrement of the bosentan orientational variety leads to a loss of configurational entropy of bosentan, another thermodynamic quantity should compensate the free-energetic shortcoming by the decrement of the orientational variety. Otherwise, the density of the bosentan centroid, $\rho_{CMb} \left( {{\varvec{r}}_{cube} } \right)$, should decrease in the binding pocket. Our results indicate that the intermolecular native contacts (electronically attractive interactions) between bosentan and hETB are formed in the binding pocket (Fig. 9). It is likely that this native contact formation is the compensation factor, and that, eventually, the native-complex conformation is completed.

Results obtained from the present study suggest that a longer N-terminal tail adds a new property to the binding mechanism. Moreover, it presents the possibility that the binding speed might be enhanced by designing the length and sequence of the N-terminal tail. In fact, earlier reports describe that long N-terminal tails exert various physiological functions of GPCRs such as ligand recognition, receptor activation, and signaling^22,23^. Therefore, a simulation with a longer N-terminal tail is the next challenge for ligand–GPCR simulation.

We uploaded important snapshots obtained from the current simulation to the Biological Structure Model Archive (BSM-Arc)^63^. The entry for this study is https://bsma.pdbj.org/entry/35.

## Supplementary Information

Supplementary Information.

## Data Availability

The datasets used or analyzed during the current study are available from the corresponding author on reasonable request. Several important snapshots during the current simulation were available in the BSM-Arc (https://bsma.pdbj.org/entry/35).
